# Uncovering a hidden functional role of the XRE-cupin protein PsdR as a novel quorum-sensing regulator in *Pseudomonas aeruginosa*

**DOI:** 10.1371/journal.ppat.1012078

**Published:** 2024-03-14

**Authors:** Huifang Qiu, Yuanhao Li, Min Yuan, Huali Chen, Ajai A. Dandekar, Weijun Dai

**Affiliations:** 1 Integrative Microbiology Research Center, College of Plant Protection, South China Agricultural University, Guangzhou, China; 2 Department of Microbiology, University of Washington, Seattle, Washington, United States of America; University of Massachusetts Chan Medical School, UNITED STATES

## Abstract

XRE-cupin family proteins containing an DNA-binding domain and a cupin signal-sensing domain are widely distributed in bacteria. In *Pseudomonas aeruginosa*, XRE-cupin transcription factors have long been recognized as regulators exclusively controlling cellular metabolism pathways. However, their potential functional roles beyond metabolism regulation remain unknown. PsdR, a typical XRE-cupin transcriptional regulator, was previously characterized as a local repressor involved solely in dipeptide metabolism. Here, by measuring quorum-sensing (QS) activities and QS-controlled metabolites, we uncover that PsdR is a new QS regulator in *P*. *aeruginosa*. Our RNA-seq analysis showed that rather than a local regulator, PsdR controls a large regulon, including genes associated with both the QS circuit and non-QS pathways. To unveil the underlying mechanism of PsdR in modulating QS, we developed a comparative transcriptome approach named “transcriptome profile similarity analysis” (TPSA). Using this TPSA method, we revealed that PsdR expression causes a QS-null-like transcriptome profile, resulting in QS-inactive phenotypes. Based on the results of TPSA, we further demonstrate that PsdR directly binds to the promoter for the gene encoding the QS master transcription factor LasR, thereby negatively regulating its expression and influencing QS activation. Moreover, our results showed that PsdR functions as a negative virulence regulator, as inactivation of PsdR enhanced bacterial cytotoxicity on host cells. In conclusion, we report on a new QS regulation role for PsdR, providing insights into its role in manipulating QS-controlled virulence. Most importantly, our findings open the door for a further discovery of untapped functions for other XRE-Cupin family proteins.

## Author summary

*Pseudomonas aeruginosa* is a leading cause of opportunistic acute and chronic infections in humans. *P*. *aeruginosa* uses quorum-sensing (QS) to regulate gene expression in response to cell density, and QS in *P*. *aeruginosa* has been implicated in a variety of group behaviors of this bacterium, including the regulation of virulence functions. However, the regulatory mechanisms governing QS in *P*. *aeruginosa* remain incompletely understood. Here, we describe a function for PsdR, a member of the XRE-Cupin family of transcriptional regulators, as a negative regulator of *P*. *aeruginosa* QS. PsdR does so by directly and negatively regulating expression of the QS transcription factor LasR. This regulation of *lasR* expression is a novel mechanism for regulation of *P*. *aeruginosa* QS and implicates PsdR as a negative regulator of virulence in *P*. *aeruginosa*.

## Introduction

*Pseudomonas aeruginosa* is an opportunistic pathogen that causes severe acute and chronic human infections, particularly in cystic fibrosis (CF) patients with compromised immune systems [1,2]. A cohort of virulence factors in *P*. *aeruginosa* is under the control of quorum-sensing (QS) [3]. QS, a cell-cell communication system, regulates the expression of hundreds of genes and coordinates bacterial behavior in a cell-density-dependent manner [4]. The QS circuit of *P*. *aeruginosa* consists of two complete acyl-homoserine lactone (AHL) systems, LasR-I and RhlR-I. In the Las QS system, LasI catalyzes the production of the diffusible QS signal N-3-oxododecanoyl homoserine lactone (3OC12-HSL), which binds to the transcriptional regulator LasR. The active 3OC12-HSL-bound LasR then activates the expression of dozens of downstream genes. Similarly, in the Rhl QS system, RhlI synthesizes butyryl-HSL (C4-HSL) and the C4-HSL-bound RhlR activates the expression of Rhl-regulon [5]. These two AHL QS systems interweave with a third QS system, the *Pseudomonas* quinolone signal (PQS) system, which involves the generation of 2-heptyl-3-hydroxy-4-quinolone (PQS) and its precursor 4-hydroxy-2-heptylquinoline (HHQ) that bind to the signal receptor PqsR [6,7]. In general, QS systems in *P*. *aeruginosa* are organized hierarchically, with the Las QS system atop the QS hierarchy, and thus the inactivation of either LasI or LasR leads to the inactivation of all three QS circuits [3].

QS is characterized by high energy expenditure. Therefore, checks on its positive feedback loop are crucial to maintain an optimal level of QS activity. In *P*. *aeruginosa*, a suite of QS regulators play a crucial role in providing a negative homeostasis to Las QS [4]. One of them is RsaL, a transcriptional repressor that directly binds to the promoter of *lasI*, resulting in the inhibition of both *lasI* and *lasR* expression [8]. Interestingly, expression of RsaL was found to be under the control of LasR [9], indicating a reciprocal regulation between RsaL and LasR. Another regulatory protein, AlgR2, was identified as a negative modulator for *lasR* and *rhlR* gene expression [10]. In addition, three anti-activators QscR, QslA and QteE, which control the QS activation threshold have been identified. QscR, an orphan LuxR homologue, somehow alters the timing and amount of signal produced [11,12]. QteE acts as an anti-activator by reducing the stability of the LasR protein without affecting its transcription or translation [13]. Similarly, QslA inhibits LasR by binding to it through protein-protein interaction, thus disrupting LasR dimerization [14,15]. These identified anti-activators directly target the LasR protein, permitting negative control of the Las QS system.

The *P*. *aeruginosa* genome encodes at least 8 transcriptional regulators that contain an N-terminal helix-turn-helix xenobiotic response element (XRE) domain and a C-terminal cupin sensor domain [16]. The XRE domain allows the interaction of the protein with DNA targets, while the cupin sensor domain enables the bacterium to sense signal molecules. These XRE-cupin proteins were initially described to form a functional unit with neighboring genes, and inhibit the expression of these genes involved in condition-specific metabolic pathways [16,17]. Each XRE-cupin member has its own DNA targeting specificity, and no common target genes among these regulators could be identified. One prominent example of an XRE-cupin member is PsdR, which binds to DNA motifs containing a palindromic sequence in a dimeric structure [16,17]. Like other characterized XRE-cupin transcriptional regulators, PsdR was described as a local transcriptional regulator, repressing the transcription of its two neighboring genes, *mdpA* and *dppA3* [16]. Notably, *mdpA* and *dppA3* encode a metallo-dipeptidase [17] and a small peptide transport [18], respectively. Through transcriptional repression of *mdpA* and *dppA3*, PsdR acts as a dipeptide regulator, governing the transport and processing of dipeptides in *P*. *aeruginosa* [16,17,19].

Mutations in the *psdR* gene were commonly observed in the laboratory strain PAO1 of *P*. *aeruginosa* when it underwent experimental evolution in a minimal medium, in which casein serves as the sole carbon and energy source [19,20]. In this environment, the growth of *P*. *aeruginosa* requires QS activation, as QS-controlled extracellular proteases are needed for the degradation of casein. Asfahl et al. described a non-social role of PsdR mutants in this QS-active evolution process, independent of QS circuit regulation [19]. In casein broth, spontaneous *psdR* mutations quickly emerge and become fixed throughout the entire population, occurring much earlier than LasR mutant cheaters [19,20]. The selection for PsdR mutants within an evolving population indicates that it possesses a relative growth advantage compared to the subpopulation containing an intact PsdR. As described, this growth advantage was attributed to the role of PsdR in regulating the metabolism of dipeptides—mutations of *psdR* increased their processing and transport. This non-QS role of PsdR mutants resulted in an elevated tolerance of the population for the invasion of the LasR-null mutant cheaters, and thus delayed the occurrence of a tragedy of the commons [19].

In the present study, we systematically investigated the QS-dependent phenotypes and regulatory mechanisms of PsdR on QS regulation. In contrast to the non-QS role of PsdR as described before, our study unveiled PsdR as a novel negative QS transcriptional regulator, modulating QS activity independent of its known dipeptide metabolism pathway. Rather than a local transcriptional repressor, we found that PsdR likely acts as a potential global regulator, with a much larger regulon than previously described. Furthermore, in the present study we developed a streamlined bioinformatic approach for comparative transcriptome analysis with a cohort of transcriptome data sets. Using this approach, we observed that the transcriptome profile of the PsdR-expressing strain closely resembled that of the LasR-null mutant. We found that *lasR* indeed is a direct regulatory target for PsdR, and QS modulation by PsdR is LasR-dependent. We demonstrate that PsdR directly binds to the *lasR* promoter and suppresses its expression at the transcriptional level. Consequently, inactivation of PsdR results in heightened QS activity. Overall, our work uncovers PsdR as a novel regulator of QS-controlled processes, and provides insights into its mechanism of QS regulation.

## Results

### PsdR is a novel negative QS regulator in *P*. *aeruginosa*

PsdR, a typical member of the XRE-cupin family protein, is known to strictly control dipeptide utilization and transport by regulating the expression of its two neighboring genes [16,17,19]. In casein broth, where casein is the sole carbon and energy source, activation of QS is required to express proteases needed for cell growth [21,22]. When *P*. *aeruginosa* strain PAO1 is grown in casein broth, PsdR mutants quickly emerge and become fixed in the population [19]. PsdR was described to have no effect on the QS circuit [19]. However, we observed an increased production of soluble green pigments in the PsdR-null mutant when grown in casein broth (S1 Fig). These pigments were likely phenazines, which are metabolites controlled by QS in *P*. *aeruginosa* [23]. This finding suggested that PsdR may be involved in the production of QS-controlled metabolites. We therefore hypothesized that PsdR may harbor additional uncharacterized functions related to QS regulation.

**Fig 1:**
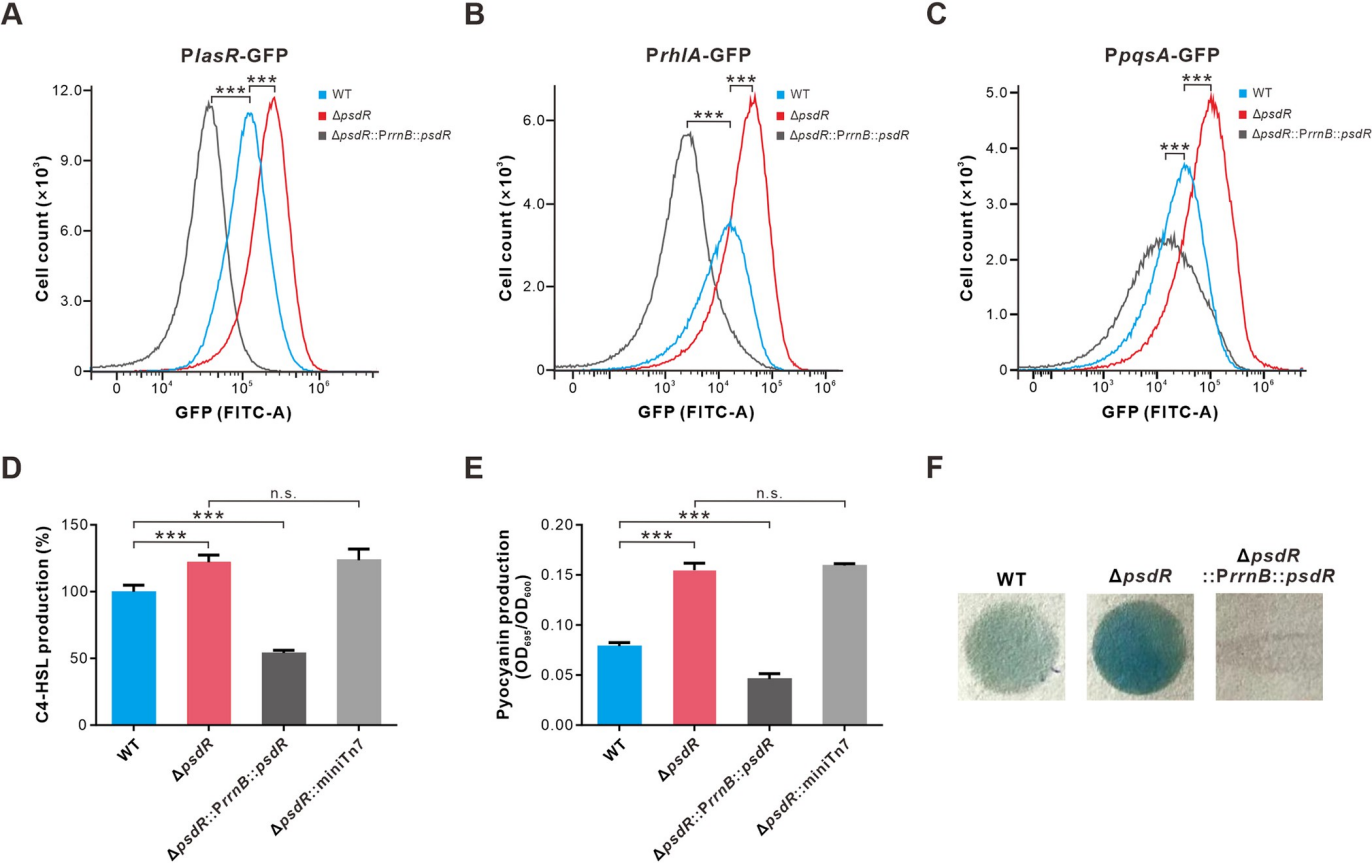
The expression of PsdR influences QS activity. (A-C) Las, Rhl and PQS activity. The activities of the Las, Rhl and PQS QS systems are reflected by the fluorescence levels of the expressed reporters P*lasR*-GFP (A), P*rhlA*-GFP (B) and P*pqsA*-GFP (C), respectively. Fluorescence values were obtained from bacteria cultured in casein broth for 12 h using a flow cytometer (FITC channel). (D-F) The relative concentrations of QS-controlled metabolites, C4-HSL (D), pyocyanin (E) and hydrogen cyanide (F) in shown strains. Production of C4-HSL in WT was set to 100%. WT, wild-type strain PAO1; Δ*psdR*, PsdR-null mutant; Δ*psdR*::P*rrnB*::*psdR*, PsdR-null mutant carrying a single copy of *psdR* driven by the *rrnB* promoter; Δ*psdR*::miniTn7, PsdR-null mutant carrying a single copy of empty miniTn7 vector. A one-way ANOVA with Bonferroni posttest was used for statistical analysis. *P < 0.05, **P < 0.01, ***P < 0.001.

To test our hypothesis, we mobilized three different QS reporter plasmids into *P*. *aeruginosa* strains that either contained or lacked the *psdR* gene. These reporter plasmids contained transcriptional fusions that reflect the activity of the Las (P*lasR*-GFP), Rhl (P*rhlA*-GFP), or PQS system (P*pqsA*-GFP). Since bacteria grown in casein broth cannot be measured by optical density due to the proteolyzed casein [24], we measured the activities of these QS reporters using flow cytometry as previously described [25]. Compared to wild-type PAO1, the fluorescence levels of QS reporters showed a pronounced increase in PsdR-null cells, while a considerable decrease was observed in PsdR-overexpressing cells (Fig 1). Similar results were also obtained when strains carrying QS reports were grown in casamino acids medium (minimal medium supplemented with casamino acids) (S2 Fig). These results of the QS reporter assay suggest that PsdR affects the expression of genes known as representatives of the QS circuit. Furthermore, we quantified a number of QS-controlled products in the PsdR derivative cells. QS-controlled metabolites, such as QS signal C4-HSL, pyocyanin and hydrogen cyanide, were also significantly elevated in PsdR-null cells. On the contrary, PsdR expression substantially reduced their biosynthesis (Fig 1). Our results demonstrate that PsdR negatively modulates QS phenotypes.

### A large PsdR-induced regulon

**Fig 2:**
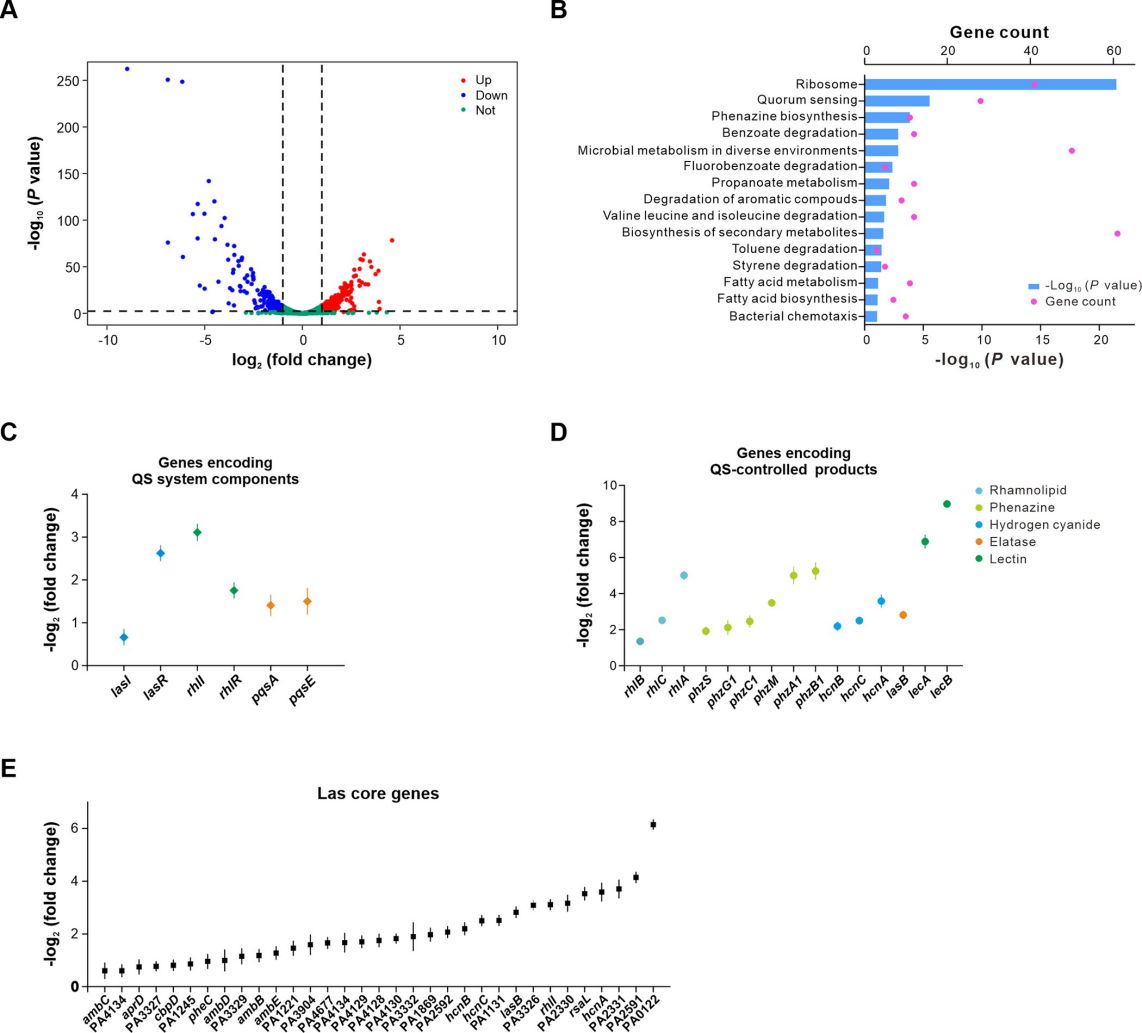
Transcriptome analysis of the PsdR-expressing cells. (A) Volcano plots showing the magnitude of differential gene expression induced by the PsdR-expressing strain (WT::P*rrnB*::*psdR*) compared to the wild-type strain PAO1. Each dot represents one annotated sequence with detectable expression. Red dots, up-regulated genes; blue dots, down-regulated genes; green dots, genes that are not differentially expressed. Thresholds for defining a significance in differential expression (log_2_ (fold change) ≧ |1.0|, *P* value ≦ 0.05) are shown as dashed lines. (B) KEGG pathway analysis of differentially expressed genes in the WT::P*rrnB*::*psdR* strain compared with wild-type strain. Gene count number and a negative log_10_ *P* value shows the enrichment of differentially expressed genes in the corresponding pathway. (C-E) A negative log_2_ (fold change) value indicates the down-regulation of genes encoding QS system components (C) involved in QS-controlled products (D) and associated with the Las core regulon [26] (E) in the WT::P*rrnB*::*psdR* strain compared with wild-type strain. WT::P*rrnB*::*psdR*, wild-type PAO1 strain carrying a single copy of *psdR* driven by the *rrnB* promoter. Data are presented as mean ± SD.

To investigate genome-wide transcriptomic changes caused by PsdR, we sought to conduct an RNA-sequencing (RNA-seq) analysis and compared the transcriptome profile of the PsdR derivative with that of the parent wild-type strain PAO1. Performing RNA-seq analysis with strains cultured in casein broth presents a challenge because non-uniform replicate samples would be obtained in this environment. Due to this technique issue, PsdR derivatives were grown in casamino acids medium. In this medium, all assayed strains had a similar growth curve (S3 Fig). Since the expression level of PsdR is relatively low in casamino acids medium compared with casein broth (S4A Fig), the PsdR-expressing strain (WT::P*rrnB*::*psdR*), genomically integrated with a single copy of *psdR* driven by the *rrnB* P1 promoter, was subjected to RNA-seq analysis in comparsion to PsdR-null cells. This approach resulted in a mild increase in *psdR* expression (S1 Table and S4B Fig). In contrast to a local, highly specialized role of PsdR as previously described [16,17], our sequencing results revealed that mild PsdR overexpression markedly altered the global transcriptome profile, with a total of 504 genes (about 8.8% of total annotated genes encoded in the genome) differently expressed (log_2_(fold change) ≧ 1.0, *P* < 0.05) (Fig 2 and S1 Table). These differentially expressed genes were enriched in various cellular pathways as presented by the KEGG mapping (Fig 2B). Notably, one of them is QS. In comparison to the transcriptome of PsdR-null cells, mRNA levels of genes controlled by the three QS systems were almost universally down-regulated in the PsdR-expressing cells, resulting in significantly decreased expression levels of genes encoding QS system components and QS-controlled products (Fig 2C and 2D). This transcriptome analysis further supported the idea that PsdR regulates the QS circuit.

We next reviewed the expression change of a core set of 42 genes, which was defined as the Las QS core regulon across seven *P*. *aeruginosa* isolates from distinct resources [26]. Under the condition of PsdR expression, 78.6% (33/42) of these Las core genes were differentially expressed. Their expression levels were significantly lower, with 75.8% (25/33) having more than a two-fold change (Fig 2E). Moreover, this Las QS core regulon was enriched for decreased gene expression (*P* = 3.2 ×10^−4^, *Chi-squared* test). In addition to QS-related genes, PsdR also induced gene expressions in those pathways not associated with QS circuit and dipeptide metabolism (S1 Table). Overall, our RNA-seq analysis revealed a rather larger PsdR-induced regulon than previously described [16,17,19], and PsdR expression has unexpectedly more extensive impacts on genes not only involving in the QS pathway but also other regulatory circuits.

### PsdR modulates QS independent of dipeptide regulation

PsdR is known to function as a local transcriptional repressor, specifically targeting its two adjacent genes, *mdpA* and *dppA3* [16,17]. We asked the question of whether PsdR regulates QS through these two genes. To address this question, we deleted both *mdpA* and *dppA3* in the PsdR-null mutant and examined their influence on QS. Interestingly, we found that additional deletion of *mdpA* and *dppA3* had no or little consequences on QS. The production of green pigments was not affected, as observed in the PsdR-MdpA-DppA3 mutant cells grown in casein broth (S1 Fig). Furthermore, the activities of three QS reporters remained substantially enhanced in the PsdR-null mutant, regardless of the presence or absence of *mdpA* and *dppA3* (S5 Fig), indicating that neither of these two genes is linked to the QS circuit, and that the phenomenon is not due to the increased peptide transport in PsdR mutants. Finally, consistent with the results of the QS reporter, the lack of *mdpA* and *dppA3* did not affect the production of various QS-controlled products, such as pyocyanin and hydrogen cyanide (S5 Fig). Taken together, these results suggest that the known dipeptide metabolism pathway is not responsible for PsdR-mediated QS regulation. Considering the large PsdR-induced regulon identified in our transcriptome analysis (Fig 2), we hypothesized that PsdR may directly target other genes for QS modulation rather than *mdpA* and *dppA3*.

### Development of a comparative transcriptome analysis approach for the identification of the regulatory mechanisms of PsdR

Given a vast amount of transcriptome data of various *P*. *aeruginosa* regulatory factors is available in public databases, we reasoned that those data may provide helpful clues for uncovering the unknown mechanism of PsdR responsible for QS regulation. In a conventional bioinformatic approach, the transcriptome data of a regulatory factor was analyzed to determine gene sets that were differentially expressed. The relevant pathways can then be found based on identified differentially expressed genes. However, this approach leads to the identification of a large number of candidate genes as well as regulatory pathways, making more difficulty in searching for potential direct target genes. We thereby developed a streamlined comparative transcriptome analysis approach, in which the transcriptome data set of a variant of regulatory factors was statistically analyzed. We assumed that if a similar transcriptome profile is found between different regulatory factors, they likely function through the same or a similar pathway. In other words, PsdR may possess a similar regulatory mechanism to other regulatory factors with known functions, if its transcriptome profile is analogous to that of those factors. This strategy allows the filtering out of irrelevant candidates, thus facilitating the identification of specific regulatory mechanisms. This method, termed “transcriptome profile similarity analysis" (TPSA), is schematically illustrated in S6 Fig.

### The TPSA method results in the identification of a LasR-null-like transcriptome profile in PsdR-expressing cells

**Fig 3:**
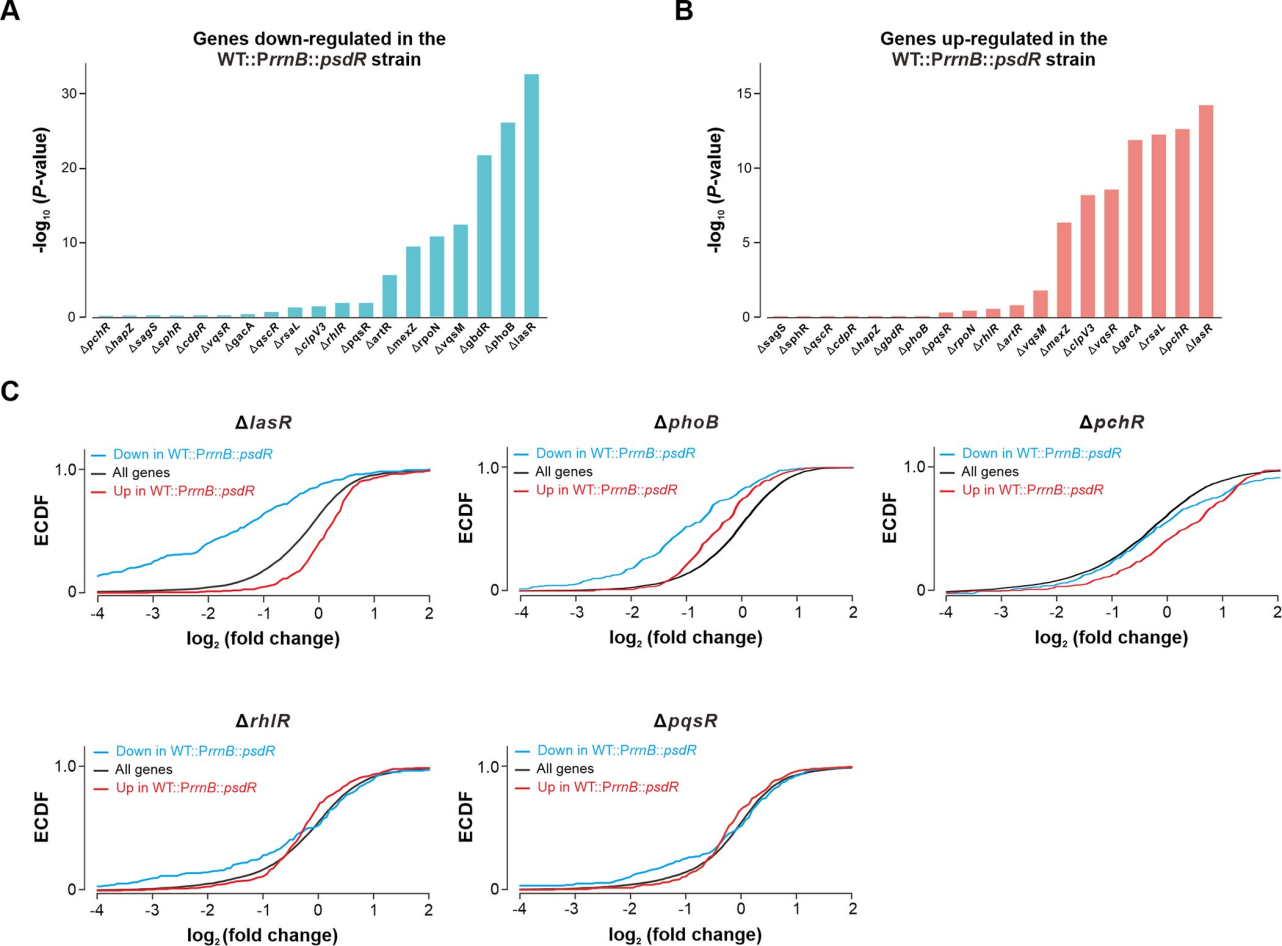
Gene expression profile in regulator transcriptomes. (A-B) Expression shift of down-regulated (A) or up-regulated (B) genes in each regulator transcriptome. Transcriptome similarity was estimated using one-sided KS test. The graph shows the corresponding negative log_10_-transformed *P* value for each regulator transcriptome. (C) Empirical cumulative distribution functions (ECDF) analysis for gene expression changes in PsdR-expressing strain compared with LasR-null, PhoB-null, PchR-null, RhlR-null and PqsR-null. Genes with a two-fold expression level change in the PsdR-expressing strain were applied to produce the plot. WT::P*rrnB*::*psdR*, wild-type PAO1 strain carrying a single copy of *psdR* driven by a *rrnB* promoter.

Using the established TPSA method, we sought to evaluate the expression pattern between PsdR and other regulatory factors. A total of 19 transcriptomic datasets from the NCBI SRA database were analyzed. These transcriptomes include both QS and non-QS regulators in *P*. *aeruginosa* strain PAO1 (S2 Table). The common gene sets differentially expressed (two-fold change as threshold) among the transcriptomes of examined regulators was first determined. The expression shift of up- or down-regulated gene sets in each regulator mutant was then estimated with its own transcriptome using the Kolmogorov-Smirnov test (KS test). This strategy allows us to examine the expression patterns of various transcriptomes conducted in different conditions. The similarity of expression pattern between PsdR and each examined regulator was ranked according to their log-transformed *P* values. Using this method, we found that mutants of LasR and PhoB obtained the smallest *P* values when estimating expression changes of down-regulated gene sets in PsdR-expressing cells (Fig 3A). This result indicates a similar expression pattern for these gene sets observed in PsdR-expressing cells to that in the LasR-null or PhoB-null backgrounds. PhoB is a response regulator of the two-component regulatory system PhoR-PhoB, and acts as a positive QS regulator, activating QS circuits by competitively binding to the promoter of *lasI* against LasR and RsaL [27]. On the other hand, PsdR-expressing cells were most similar to the LasR-null and PchR-null mutants with respect to the expression pattern for up-regulated gene sets (Fig 3B). PchR is an AraC-type transcriptional activator involved in siderophore regulation [28], and so far its association with QS circuits has not been reported in the literatures. Overall, the transcriptome in LasR-null is most similar to that of PsdR-expressing cells as analyzed by our TPSA method. In conclusion, our findings indicate that PsdR-expressing cells displayed a LasR-null-like transcriptome. Based on these observations, we reasoned that PsdR may modulate the QS circuit through inhibition of the Las QS system.

To survey the potential direct interaction of PsdR with Rhl and PQS QS systems, we examined the transcriptomes in RhlR-null and PqsR-null mutants and compared them to that in PsdR-expressing cells. As shown by the empirical cumulative distribution functions (ECDF) plot analysis, PsdR-induced expression changes in those gene sets were likewise observed in LasR-null for both down- and up-regulated gene sets, while in PhoB-null and PchR-null only for down- and up-regulated gene sets, respectively (Fig 3C). However, this expression pattern was not observed in either the RhlR-null or PqsR-null backgrounds. These combined results suggest that PsdR induced a transcriptome similar to LasR, but distinct from RhlR or PqsR. Taken together, the application of the TPSA method in the present study provides clues that PsdR may primarily interact with the Las QS system rather than the Rhl or PQS QS systems.

### Confirmation of LasR-null-like phenotypes in PsdR-expressing cells

Based on the results obtained from our TPSA analysis, we expected that PsdR-expressing cells may exhibit LasR-null-like QS phenotypes. We therefore examined the QS-controlled products in these cells. The productions of pyocyanin, elastase and hydrogen cyanide, which are virulence factors of *P*. *aeruginosa* and known to be regulated by LasR [29], were measured in PsdR-expressing cells and compared to that in the LasR-null mutant. As expected, the production of these QS products in PsdR-expressing cells was reduced to the same level as LasR-null cells (S7 Fig). Overall, PsdR-expressing cells exhibited a typical LasR-null-like phenotype in terms of the biogenesis of various virulence factors. Of particular note, PsdR-expressing cells deficient in these QS-controlled virulence factors also displayed a cell killing level similar to that observed in LasR-null cells, as demonstrated by the release of cytosolic lactate dehydrogenase (LDH) experiment with host Chinese hamster ovary (CHO) cells (Fig 7). In summary, cells expressing PsdR exhibited a LasR-null-like transcriptome and corresponding phenotypes.

### The *lasR* gene is a direct regulatory target for PsdR

**Fig 4:**
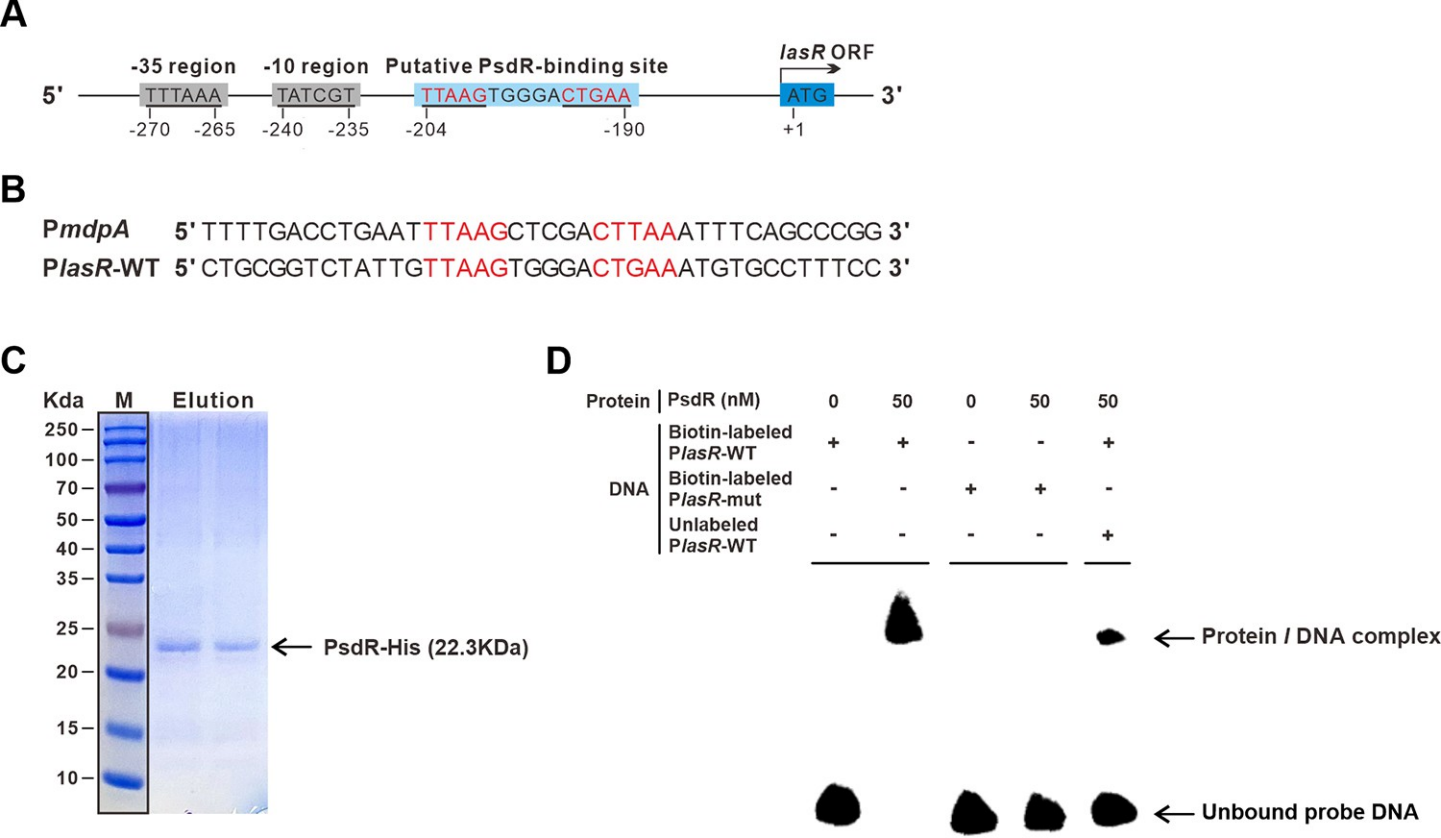
PsdR directly binds to the promoter region of *lasR*. (A) Schematic diagram of the promoter region of the *lasR* gene. Cis-acting elements are colored. The arrow indicates the translation start site. Putative PsdR-binding sites are highlighted in red. (B) Sequence alignment of the promoter regions of *mdpA* and *lasR*. (C) SDS-PAGE of purified His-tagged PsdR protein. (D) EMSA results showing that the his-tagged PsdR protein and the probe DNA containing putative PsdR-binding site formed a protein-DNA complex. This protein-DNA complex was disrupted when the putative PsdR-binding site was deleted in the probe DNA. P*lasR*-WT, promoter DNA of the *lasR* gene containing the putative PsdR-binding site; P*lasR*-mut, *lasR* probe DNA deleting the putative PsdR-binding site. Probe DNA was either 3’end biotin-labeled or unlabeled. The protein-probe complex and unbound DNA probe and are indicated by arrows. The experiment was independently performed more than three times and obtained data were similar.

Based on the results of the TPSA analysis, we hypothesized that PsdR might directly interact with and repress one or more elements of the Las QS system. As a first step in testing our hypothesis, we first analyzed the promoter regions of *lasI* and *lasR* for potential PsdR-binding sites. Previous studies identified a palindromic sequence (5’-TTAAGxxxxxCTTAA-3’, x indicates a nucleotide space) in both the promoter regions of *mdpA* and *dppA3* [16]. PsdR recognizes and binds to this palindromic sequence in a dimer structure, leading to the repressed expression of both these genes. However, such PsdR-binding sites could not be found in the region of the *lasI* promoter. Meanwhile, the transcription level of *lasI* was not substantially altered by the expression of PsdR in our transcriptome analysis (Fig 2C). Therefore, it is very unlikely that *lasI* is a direct regulatory target for PsdR. We next turned to examine the promoter region of *lasR* with respect to potential PsdR-binding sites. In fact, a similar short non-palindromic sequence (5’-TTAAGxxxxxCTGAA-3’) was identified in the *lasR* promoter region, with only one nucleotide difference from the known PsdR-binding site (Fig 4A and 4B). Furthermore, a more than 6-fold transcriptional change of *lasR* was observed by the induction of PsdR (Fig 2C). All these data pointed to the possibility that PsdR might directly regulate *lasR* expression through binding to this putative binding site.

Encouraged by the identification of a putative PsdR-binding site within *lasR* promoter, we conducted an electrophoretic mobility shift assay (EMSA) to investigate whether PsdR could bind to it. In this EMSA experiment, a DNA fragment containing the wild-type *lasR* promoter region was used as an assay probe, and the same DNA fragment with deletion of the putative PsdR-binding site was employed as a control probe. A his-tagged version of the PsdR protein was expressed and purified from engineered *Escherichia coli* cells (Fig 4C). Our EMSA results showed that this His-tagged PsdR protein bound to the *lasR* probe DNA, resulting in a slower migration for the *lasR* probe in the gel (Fig 4D). In contrast, this DNA migration could not be observed when the PsdR protein was applied with the control probe lacking the putative PsdR-binding site (Fig 4D). These results of EMSA in our study provide evidence that PsdR binds to the identified putative binding site in the *lasR* promoter, demonstrating its capacity to recognize and interact with a non-palindromic sequence. In conclusion, our findings showed that in addition to the known *mdpA* and *dppA3* genes [16,17,19], *lasR* is a new direct regulatory target for PsdR.

### PsdR modulates the QS circuit through direct regulation of *lasR* expression

**Fig 5:**
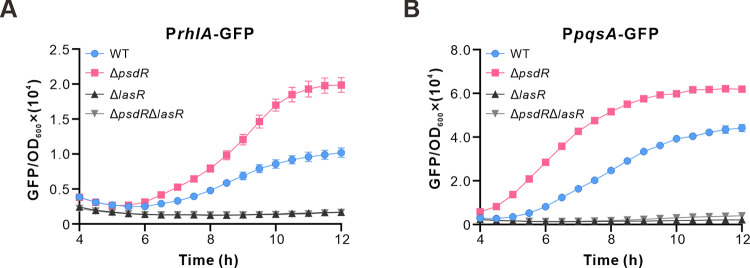
PsdR modulates QS activity in a LasR-dependent manner. (A-B) Evaluation of respective Rhl and PQS QS activity in shown strains by using P*rhlA*-GFP (A) and P*pqsA*-GFP (B) reporter plasmids. Strains were grown in casamino acids medium. Fluorescence values were determined from the following strains: WT, wild-type PAO1 strain; Δ*psdR*, PsdR-null mutant; Δ*lasR*, LasR-null mutant; Δ*psdR*Δ*lasR*, double deletion mutant. Data are means ± SD from a representative experiment (*n* ≧ 4). In some cases, the error bars are too small to be seen.

Our results of TPSA and EMSA led us to reason that PsdR may regulate the QS circuit by directly targeting LasR. To test this assumption, we began with the deletion of *lasR* from the PsdR-null mutant, generating the mutant PsdR-LasR. QS reporter constructs were then mobilized into these PsdR derivative mutants and their activities were examined. Fluorescent signals in the PsdR-LasR mutant carrying QS reporter constructs were dramatically decreased, reaching to a level similar to that in the LasR-null mutant carrying QS reporter constructs (Fig 5). This result demonstrates that QS modulation by PsdR relies on its regulation of the *lasR* expression. We further inferred that PsdR may indirectly affect the LasR regulon. Specifically, we examined the expression level of PA4677, a gene known to be specifically controlled by LasR but not RhlR [30]. As expected, we found that the expression level of PA4677 was negatively correlated with the expression of PsdR, as reflected by the examination of the activity of its promoter-GFP fusion construct (S8 Fig). Taken together, our results reveal that PsdR modulates the QS circuit through direct regulation of the *lasR* expression.

### PsdR alters the QS activation threshold by controlling *lasR* transcription

**Fig 6:**
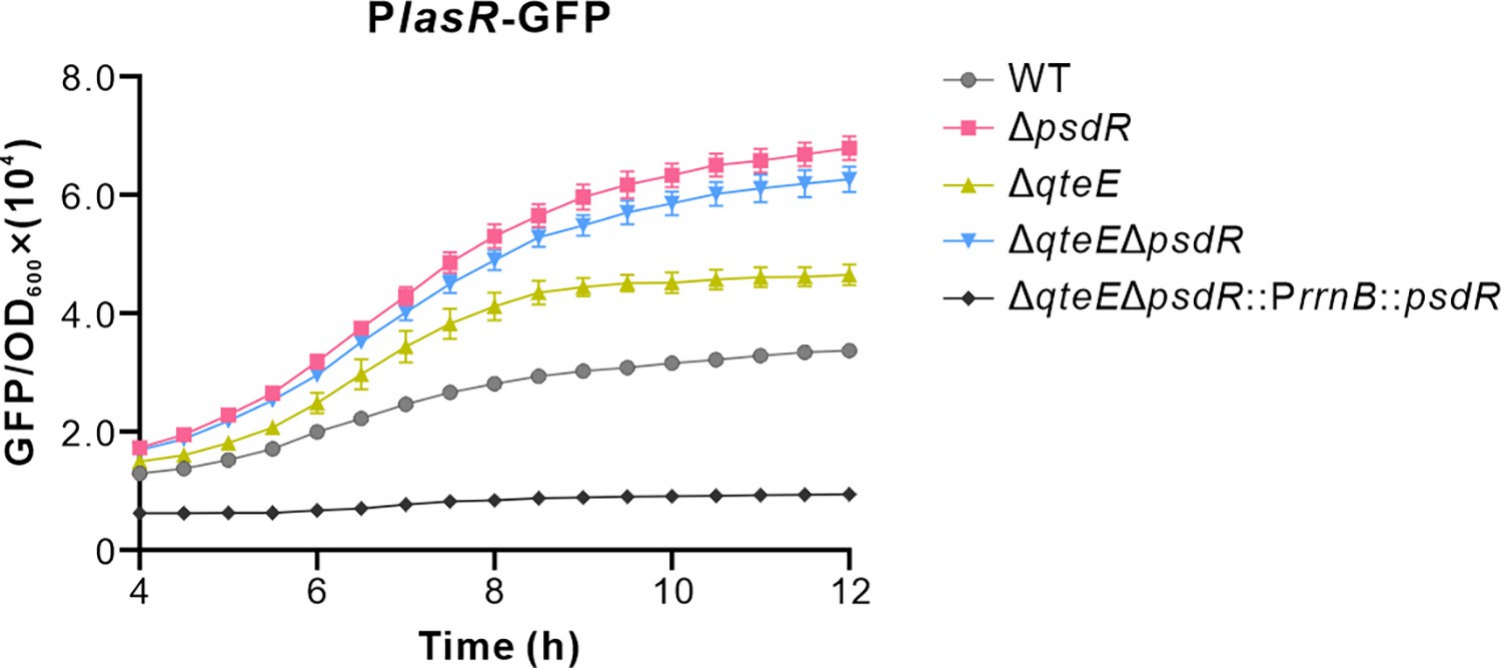
PsdR expression affects QteE-mediated LasR regulation. The expression levels of *lasR* reflected by the P*lasR*-GFP report plasmid in shown strains. These strains were grown in casamino acids medium. WT, wild-type PAO1 strain; Δ*psdR*, PsdR-null mutant; Δ*qteE*, QteE-null mutant; Δ*qteE*Δ*psdR*, *qteE* and *psdR* double deletion mutant; Δ*qteE*Δ*psdR*::P*rrnB*::*psdR*, *qteE* and *psdR* double deletion mutant containing a single copy of *psdR* driven by a *rrnB* promoter. Data are means ± SD from a representative experiment (*n* ≧ 4).

The government of the QS activation threshold in *P*. *aeruginosa* relies on a number of anti-activator factors, such as QslA [14,15], QscR [11,12] and QteE [13]. Because our work implicates PsdR as a transcriptional inhibitor of *lasR*, we wondered how PsdR might interact with these regulators of QS activation threshold. We started by deleting the *qteE* gene, which encodes a QS-activator that directly interacts with LasR protein [13]. Using a *lasR* promoter-GFP (P*lasR*-GFP) fusion reporter, we measured the *lasR* transcription in QteE derivative mutants in the presence or absence of PsdR. LasR expression was increased, as previously reported [13], in the QteE-null background (Fig 6). Subsequent deletion of QteE in the PsdR-null background considerably enhanced the LasR expression level, similar to that determined for the PsdR-null alone (Fig 6). This suggest the influence of QteE on LasR is marginal when PsdR is absent. However, the overexpression of PsdR resulted in dramatically reduced *lasR* transcription in the QteE mutant background (Fig 6), consistent with our findings that PsdR is a transcriptional regulator of *lasR*, and the current view that QteE affects the stability of LasR in the protein level [13]. In this regard, PsdR regulates the QS activation threshold by strictly controlling transcription of *lasR*.

### PsdR is a negative virulence regulator

We asked if PsdR mutants might also be relevant in clinical *P*. *aeruginosa* isolates that experience different selection pressures and undergo evolutionary adaptations. We conducted a search in the NCBI Protein database to retrieve PsdR protein sequences from various sources of *P*. *aeruginosa* isolates. A total of 463 PsdR protein sequences were obtained and subsequently compared with the reference sequence (NP_253189.1) of strain PAO1. Among these sequenced PsdR sequences, 97 PsdR sequences (21.0%) displayed amino acid changes, including substitution, insertion and deletion (S3 Table). Notably, the majority of these PsdR mutants (67.0%) were found in clinical *P*. *aeruginosa* isolates obtained from hospital human samples, such as sputum, blood and lung, with only 9 isolates (9.3%) originating from environments, such as soil land and river. These findings indicate a high frequency of PsdR mutants in clinical *P*. *aeruginosa* isolates, and suggest that PsdR mutants might have been selectively favored in terms of bacterial pathogenicity.

**Fig 7:**
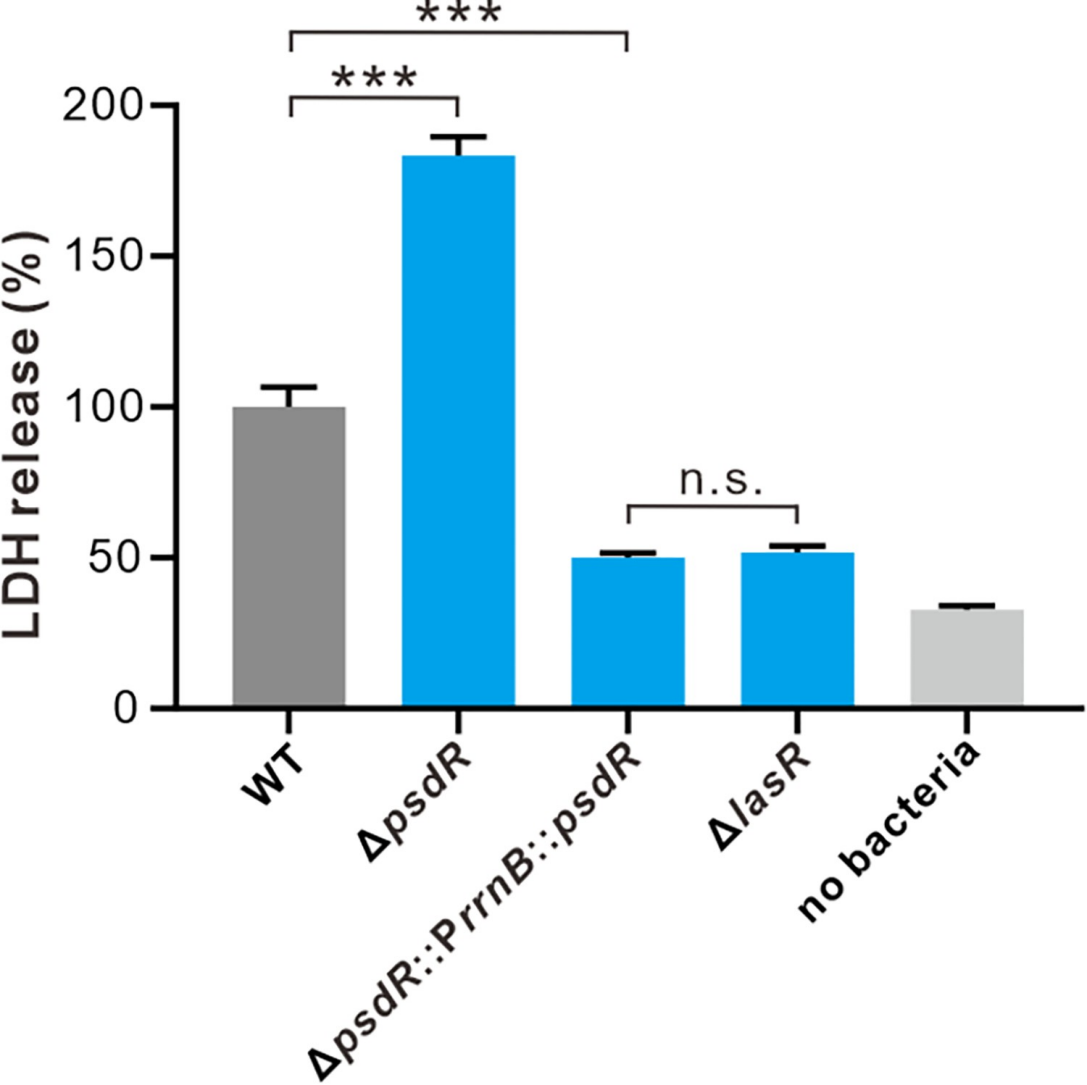
PsdR inactivation enhances host cell cytotoxicity. The killing assay with human lung cancer Chinese hamster ovary (CHO) cells was performed with equal amounts of the indicated strains. After incubation for 6 h, the release of cytosolic lactate dehydrogenase (LDH) from infected cells was quantified. The released amount of LDH inoculated with the wild-type strain (WT) was set to 100%. A one-way ANOVA with Bonferroni posttest was used for statistical analysis. *P < 0.05, ** *P* < 0.01, *** *P* < 0.001.

To investigate the impact of PsdR on bacterial pathogenicity, we measured the ability of the PsdR-null mutant to kill eukaryotic cells, in comparison to the wild-type. CHO cell line was exposed to assayed PAO1 strains, and the extent of host cell death was measured by quantifying the released LDH. In line with the induced increased production of QS-controlled virulence factors (Figs 1 and 2), the PsdR-null mutant caused significantly higher LDH release in CHO cells when compared with the wild-type strain. This observation suggests an increased level of cell death in CHO cells (Fig 7). We therefore conclude that PsdR acts as a virulence regulator that negatively influences bacterial pathogenicity, and inactivation of PsdR leads to enhanced cytotoxicity on host cells.

## Discussion

**Fig 8:**
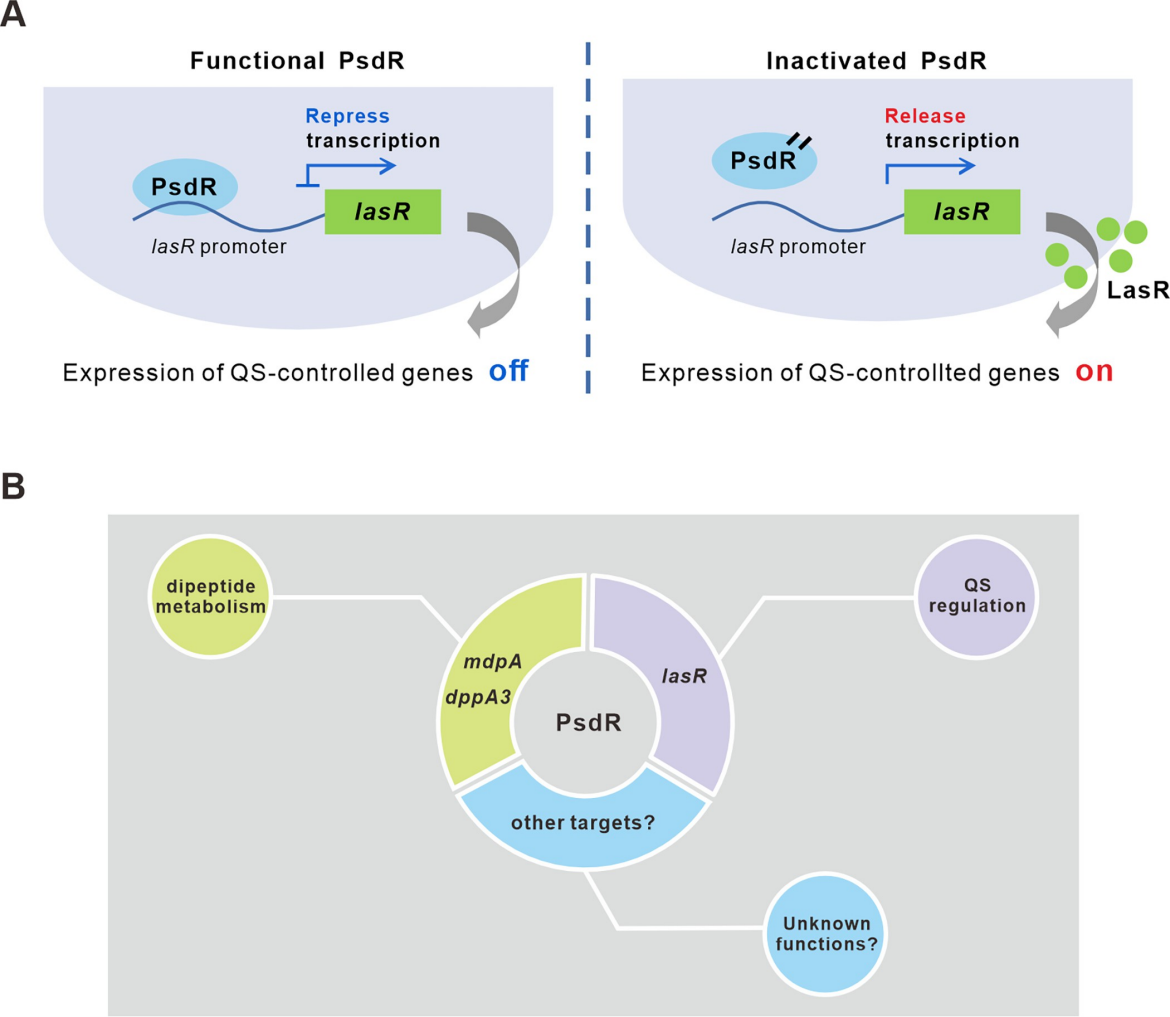
Diagrams summarizing regulatory mechanisms and functions of PsdR. (A) Diagrams illustrating that the *lasR* expression levels are regulated by PsdR and the effects on downstream gene expression. (B) The identified direct target genes of PsdR and their associated biological functions.

In the present study, we uncovered a novel function for PsdR, demonstrating that it functions as a negative QS regulator (Fig 8). PsdR is a well-known member of the XRE-cupin family proteins, characterized by its possession of an XRE DNA-binding domain and a cupin signal-sensing domain [17,31]. In previous studies, PsdR was defined as a local transcriptional repressor, which was solely involved in the uptake and utilization of dipeptide [16,17,19]. Our findings on PsdR now extend its classical role in dipeptide regulation to a new role in QS modulation. Like PsdR, other XRE-cupin proteins in *P*. *aeruginosa* were described as local, highly specialized transcriptional regulators [16]. These proteins serve as regulatory switches, modulating metabolism pathways to adapt to niche-specific conditions. For example, PauR and PA0535 were reported to regulate polyamine and arginine metabolism pathways, respectively [16,32]. The discovery of new functions for PsdR extends the conventional knowledge on XRE-cupin transcription factors in *P*. *aeruginosa*, suggesting hitherto unexplored biological functions could also be identified in other XRE-cupin proteins. Given XRE-cupin-like transcriptional factors are diverse and differentially conserved across the *Pseudomonas* genus [16], we hypothesize that some of these factors might play a central regulatory role other than the known metabolism-related functions. Our studies now open the door for making fundamental findings in the XRE-cupin family proteins.

We developed a bioinformatics approach, named TPSA, in this study to explore unknown regulatory mechanisms of PsdR in QS regulation. We used TPSA to analyze various transcriptome datasets derived from different regulator mutants in *P*. *aeruginosa*. Unlike current transcriptome approaches that primarily focus on identifying differentially expressed genes enriched in particular cellular pathways [33], TPSA enabled us to estimate the similarity between transcriptomes regulated by different known factors. Using the established TPSA, a similar transcriptome between a PsdR-expressing strain and a LasR-null mutant was identified, suggesting that *lasR* may serve as a direct regulatory target of PsdR in modulating QS. The direct binding of PsdR to the *lasR* promoter was subsequently verified by our EMSA results. It is worth noting that in this context that it would have been impossible to identify *lasR* as a new direct target for PsdR by conventional experiment approaches, such as Chromatin immunoprecipitation (ChIP)-seq analysis. In a previous study using ChIP-seq, only two genes, *mdpA* and *dppA3*, were identified as direct regulatory targets for PsdR [16]. In that ChIP-seq experiment, other candidate genes, including *lasR*, were filtered out due to their relatively weak binding signals in their corresponding promoter regions when compared with *mdpA* and *dppA3* (about one third of that exhibited by the *mdpA* and *dppA3* promoters, see S4 Table). Therefore, the application of TPSA results in an efficient connection of a previously uncharacterized protein to a well-known regulator with established functions, providing valuable clues for further uncovering its underlying regulatory mechanisms. More generally, we suggest that TPSA can be used in a wide range of research areas for scientists to explore untapped pathways and achieve important discoveries.

Bacterial QS is an energy-intensive cellular process [34], thereby mechanisms are needed to constrain its positive feedback loop. These mechanisms include QS homeostasis [35], signal molecular degrading enzymes [36,37] and import-export pumps [38,39]. In the present study, we elucidated a previously unknown negative regulatory mechanism of the Las QS system, which employs an XRE-cupin protein PsdR to control the expression level of *lasR*. While PsdR exclusively limits the transcription of the *lasR* gene, another previously identified Las negative regulator RsaL specifically targets the expression of *lasI* [8]. By placing itself under the control of LasR, RsaL provides a homeostasis feedback loop for the Las QS system [9]. Thus, protein levels of RsaL and PsdR may affect the expression levels of LasI and LasR, modulating Las QS activity. In addition to PsdR and RsaL, other regulators negatively controlling the Las QS system have also been reported in the literatures, such as QS anti-activators. QS anti-activators function to reduce the induction threshold of QS activation and delay the expression of downstream target genes [40]. QteE, QslA and QscR are thus far three identified anti-activators of the Las QS system in *P*. *aeruginosa* [11–15]. Unlike PsdR, which provides a negative regulation of LasR at the transcription level, these known QS anti-activators regulate Las QS activity by targeting the LasR protein at the post-transcriptional level. In conclusion, our work shows that PsdR regulates the Las QS at a very early stage by governing the *lasR* transcription. Together with other identified negative mechanisms, these identified negative QS regulators elegantly modulate the efficiency of the Las QS system at various physiological levels, ensuring an optimal response to environmental cues.

Transcription factors of XRE-cupin family in *P*. *aeruginosa* were supposed to have coevolved with their neighboring genes through horizontal gene transfer, forming a local functional unit [16]. An exception is PauR, a XRE-cupin-like transcription factor in *P*. *aeruginosa*, which was found to directly bind to the promoter region of 29 target genes [16,32]. Nevertheless, the function of PauR was described to be restricted in polyamine catabolism [32]. In prior studies, PsdR was found to regulate the dipeptide metabolism by controlling the transcription of its two adjacent genes [16,17,19]. In contrast, the results of our study revealed that PsdR not only regulates the known genes *mdpA* and *dppA3* but also can target the QS master regulator gene *lasR*. Consequently, the PsdR regulon identified in our study extends beyond its neighboring genes and is much larger than previously described. Moreover, the identification of a non-palindromic binding site for PsdR in the *lasR* promoter (Fig 4B) indicates that PsdR has a flexible preference for DNA recognition, implicating the possibility of a broader range of direct targets for PsdR. In support of this speculation, our transcriptome analysis demonstrated that PsdR induced a genome-wide expression change. It is worthwhile mentioning that our transcriptome analysis revealed that some of differentially expressed genes induced by PsdR were enriched in those regulatory pathways involved in neither dipeptide metabolism nor the QS circuit. Taken together, our results in the present work suggest PsdR as a potential global regulator rather than a local regulator as described before. Our findings raise the possibility of a broader regulon for other PsdR-like XRE-cupin family proteins. Future research is needed to investigate other potential direct regulatory targets of PsdR and explore the regulon specificity of other XRE-cupin proteins. These investigations may result in the identification of their hitherto unknown target genes and shed light on relevant unexplored biological functions (Fig 8B).

As a typical XRE-cupin protein, PsdR uses its cupin domain to detect small signal molecules and XRE domain to detach DNA-binding sites, through which it regulates the expression of target genes in response to particular environment cues [31]. Each cupin domain of the XRE-cupin family proteins possesses a distinct amino acid structure that forms a specific signal-sensing pocket. This structural variation determines the sensing specificity of the signal molecule for each XRE-cupin protein [16]. In other words, distinct small molecule specifically targets an XRE-cupin protein. For example, PauR, a XRE-cupin protein in *P*. *aeruginosa*, derepresses the expressions of PauR-regulated genes in response to diamines putrescine and cadaverine [32]. Similarly, PuuR, a homolog of PauR in *E*. *coli*, reduces its binding to target promoters upon increasing concentrations of putrescine [41]. Taking into account this unique feature of XRE-cupin proteins, we hypothesized that a PsdR-specific sensing small molecule might be utilized to manipulate PsdR-mediated Las QS activity and the corresponding QS-controlled pathogenicity in *P*. *aeruginosa*.

Our work uncovers a new role of the transcriptional regulator PsdR in controlling QS activity through direct repression of the QS master regulator gene *lasR*. Our findings demonstrate that PsdR inactivation may be required for a full QS activation in specific environments. PsdR inactivation not only increased the production of QS-controlled virulence factors, but also enhanced bacterial pathogenicity, as shown by the elevated cytotoxic effects on host cells (Fig 7). In addition, the high conservation of PsdR across *P*. *aeruginosa* strains [16] prompted us to hypothesize that *psdR* mutations could potentially serve as an evolutionary adaptation during *P*. *aeruginosa* clinical infections. In support of this idea, we found that *psdR* mutations are common in *P*. *aeruginosa* clinical isolates. Clinical *P*. *aeruginosa* isolates are known to thrive in stressful environments characterized by osmotic stress [42] and nitrosative stress [43]. Consequently, populations undergo evolutionary adaptations to survive in such conditions. These adaptations were found to commonly occur in genes encoding key transcriptional regulators, such as *lasR*, *gacS*, *restS*, *exsD*, *mexT*, *ampR* and *rpoN* [44–48]. Similar to *psdR*, most of these genes code for global regulators, falling into various functional categories. Studying the interplay between PsdR and these global regulators would deepen our understanding on the adaptation underlying bacterial pathogenesis. Furthermore, such studies would provide valuable insights into connections between phenotypic diversity and genetic adaptations in bacterial populations.

## Materials and methods

### Bacterial strains and growth

*P*. *aeruginosa* was grown at 37°C in Luria-Bertani (LB) broth containing 10 mg/ml tryptone, 5 mg/ml yeast extract and 10 mg/ml NaCl. LB broth was buffered with 50mM 3-(N-morpholino) propanesulfonic acid, pH 7.0 (LB-MOPS broth). In some experiments, *P*. *aeruginosa* was grown on M9 medium [22] supplemented with 1% sodium caseinate (C8654, Sigma-Aldrich, New Zealand) (casein broth) or 0.5% casamino acids (A100851, Sangon Biotech, Shanghai, China) (casamino acids medium) as the sole source of carbon and energy. *Escherichia coli* was grown in LB broth at 37°C. Unless otherwise specified, bacteria were grown in 14-mm FALCON tubes containing 3 ml of medium, with shaking (250 rpm) at 37°C. Colonies were grown in LB agar or *Pseudomonas* Isolation agar (PIA) (1.5% agar). All experiments are conducted in the *P*. *aeruginosa* strain PAO1 background. Bacterial strains used in this study are listed in S5 Table.

### Construction of *P*. *aeruginosa* mutants

Gene deletions were generated using a homologous recombination exchange approach as described previously [49]. Briefly, 500 bp DNA flanking of the target gene were PCR-amplified and cloned into the pGEX2 vector (Gentamycin resistance, Gm) [49,50] with the Vazyme ClonExpress II One Step Cloning kit (Vazyme Biotech, Nanjing, China), generating pGEX2-flanking constructs. The pGEX2-flanking construct was mobilized into the *P*. *aeruginosa* strain by triparental mating with the *E*. *coli* PRK2013 strain (Kanamycin resistance, Km). Deletion mutants were initially selected on PIA containing 100 μg/ml Gm and counter selected on LB agar containing 10% sucrose. All mutants were confirmed by PCR amplification and subsequent DNA sequencing. Primers used in this study are listed in S6 Table.

### PsdR expression

The *psdR* ORF was fused with *E*. *coli* ribosomal *rrnB* P1 promoter [51] and cloned into pUC18T-mini-Tn7T-Gm (NCBI accession number: AY599232) [52], generating miniTn7-P*rrnB*-*psdR*. P*rrnB*-*psdR* was integrated into the neutral *att* site of the genome of PAO1 with the transformation of helper plasmid pTNS2 (NCBI accession number: AY884833). The integration event was confirmed by PCR amplification and DNA sequencing. The excision of Gm resistance was performed with pFLP2 plasmid (NCBI accession number: AF048702) and selected on LB agar.

### QS reporter assay

The primers used for QS reporter constructs (P*lasR*-GFP, P*rhlA*-GFP, P*pqsA*-GFP, PPA4677-GFP) are listed in S6 Table. QS reporter plasmids were transferred into PAO1 and derivatives either by electroporation or mating, and selected on the PIA plate (Gm50). PAO1 strains bearing QS reporter plasmids were grown in LB-MOPS broth (Gm50) for 12 h, then diluted to casamino acids medium (OD_600_ ≈ 0.01) and grown to mid-log phase (OD_600_ ≈ 0.1–0.4). The mid-log phase cultures were back diluted to casein broth or casamino acids medium (OD_600_ ≈ 0.005 or 0.01) and subsequently transferred to 96-well plates (200 μl/well) with eight technique replicates. The fluorescence (excitation 488 nm, emission 525 nm) and optical density (OD_600_) of the samples were recorded every 0.5 h for 12 h using a microplate reader machine (Synergy H1MF, BioTek Instruments, Winooski, VT, USA).

### Pyocyanin measurement

Overnight cultures of *P*. *aeruginosa* strains grown in LB broth were diluted into 4 ml of casamino acids medium to reach a starting OD_600_ ≈ 0.02 and continuously cultured at 37°C for 24 h. The cells were spun down at 13,000 rpm for 2 min and the supernatant was measured at OD_695_. Pyocyanin production was determined by OD_695_ / OD_600_.

### Hydrogen cyanide production

Cells grown in LB-MOPS broth overnight were diluted to OD_600_ ≈ 0.01 by fresh LB broth and continuously cultured to mid-log phase. Subsequently, cells were adjusted to OD_600_ ≈ 0.02 and then 10 μL cells were transferred into 24-well plates containing 2% peptone agar. The plate was covered with a cyanide detection paper (Grade 3 MM Chr Cellulose Chromatography Paper, 3030–861, Whatman, UK) that was soaked in the HCN detection reagent: 10 mg copper (II) ethyl acetoacetate (41489, Alfa Assar, Shanghai, China) and 10 mg 4,4’-methylenebis-(*N*, *N*-dimethylaniline) (A18466, Alfa Assar, Shanghai, China) in 2 ml chloroform and cultured at 37°C for 18–24 h.

### Elastase production

*P*. *aeruginosa* strains grown in LB broth overnight were diluted to OD_600_ ≈ 0.02 in 3 ml LB broth and then cultured with shaking at 37°C for 8 h. Cells were then spun down at 13,000 rpm for 2 min and 500 μL supernatants were transferred to a tube containing the same amount of ECR buffer (0.1M Tris-HCl, 1mM Cacl_2_, 5 mg/ml Elastin-Congo red, pH 7.2) at 37°C for 2 h. The reaction was stopped by adding 100 μL EDTA (0.12 M). The insoluble ECR was pelleted at 5,000 g for 5 min and elastase in the supernatant was determined by measuring the absorbance at 495 nm.

### Skim milk assay

Total extracellular proteolytic activity of *P*. *aeruginosa* strains was evaluated using skim milk agar plates on which the bacteria form a protease-catalyzed clearing zone surrounding each colony. Individual colonies were grown on LB agar and then spotted on the skim milk agar plates (25% (v/v) LB, 4% (w/v) skim milk, 1.5% (w/v) agar). Colonies with extracellular proteolytic activity formed clearing zones after incubation at 37°C for 24 h. The area of transparent zones reflecting extracellular proteolytic activity was quantified from captured photographs.

### Flow cytometer analysis

PAO1 strains bearing QS reporter plasmids were grown in casein broth containing 50 μg/mL gentamycin for 12 h. Cell pellets were washed with PBS and subjected to CytoFLEX flow cytometer (Beckman Coulter, USA) for GFP measurement (FITC channel, 525/40 BP) per cell. A total of properly gated 40,000 cells per estrain were counted and the mean fluorescence was calculated with at least three technique replicates.

### Quantification of C4-HSL

*aeruginosa* strains were cultured overnight in LB-MPOS broth and diluted to a starting OD_600_ ≈ 0.05. Cells were then grown in 4 ml LB broth at 37°C for 18 h. AHLs were extracted with an equal amount of ethyl acetate. Relative quantification of C4-HSL was performed using reporter bioassay, in which C4-HSL was detected by a *E*. *coli* strain containing pECP61.5 as previously described [53].

### Mammalian cell cytotoxicity assay

CHO cells were cultured in RPMI medium (Thermo Fisher Scientific, Shanghai, China) supplemented 10% FBS (Thermo Fisher Scientific, Shanghai, China), respectively at 5% CO_2_ and 37°C. Exponentially growing *P*. *aeruginosa* bacteria cultured in casamino acids medium (OD_600_ ≈ 0.5) were diluted with cell culture medium (OD_600_ ≈ 0.1) and added to near-confluent CHO cells at a starting multiplicity of infection (MOI) ratio of 5: 1. After incubation at 37°C for 6 h, the extent of cell killing was determined by quantification of the release of lactate dehydrogenase into the cell culture supernatant using the LDH Cytotoxicity Detection Kit (Beyotime, Nantong, China).

### Production and purification of PsdR protein

Full-length *psdR* was cloned into the expression vector pET-28a (Novagen, WI, USA) and mobilized into *E*. *coli* BL21 (strain DE3, Novagen, WI, USA). pET-28a contains a His tag. Cells were induced by 1 mM IPTG at 16°C for 20 h and then were pelleted at 5,000 g for 30 min. Cells were resuspended in Lysis Buffer (25 mM Tris-HCl, 500 mM NaCl, 10 mM imidazole, 5% glycerol, 0.5% Tween-20, pH 8.0). Resuspended cells were lysed with a high-pressure homogenizer (model FPG12805, Stansted Fluid Power Ltd, Essex, UK) with the parameter of 420 MPa, 4200 bar. Lysed cells were pelleted at 15,000 g for 30 min at 4°C. The soluble supernatant fraction was incubated with Ni-NTA Agarose Beads (QIAGEN, Hilden, Germany) at 4°C for 2 h. The mixture was loaded on a chromatographic column, followingly washed by Wash Buffer (25 mM Tris-HCl, 500 mM NaCl, 20/50 mM imidazole, 5% glycerol, 0.5% Tween-20, pH 8.0) and finally eluted with Elution Buffer (wash buffer containing 250 mM imidazole) and stored at -80°C. Peak fractions were assessed by SDS PAGE analysis.

### Electrophoretic mobility shift assay (EMSA)

The *lasR* promoter sequence was amplified by PCR (primers seen in S6 Table) and used as a DNA probe (P*lasR*-WT). The putative PsdR-binding sties were removed to generate a negative control probe (P*lasR*-mut). The probe DNA was labeled with biotin Biotin-11-UTP (Jena Bioscience, Jena, Germany) using Terminal Deoxynucleotidyl Transferase (TakaRa, Dalian, China). The labeled probe (30 ng) was incubated with 50 nM purified PsdR in Binding Buffer (10 mM Tris-HCl, 50 mM KCl, 1 mM DTT, 1% glycerin,10 mM MgCl2,1 μg/μl Poly (dI•dC), pH 7.5) at 25°C for 30 min. For the competition, 50-fold unlabeled DNA probe were incubated with the labeled probe. DNA-protein complex was subjected to electrophoresis on 6.5% polyacrylamide gel. DNA subsequently was transferred to a Hybond-N+ nylon membrane (Amersham, Braunschweig, Germany) and incubated with milk Blocking Buffer (50 g/L milk powder in TBST buffer, pH 7.5) for 30 min with shaking at room temperature. The membrane was then incubated with streptavidin-horseradish peroxidase conjugate (Bioss, Beijing, China) in Blocking Buffer for 30 min at room temperature. The membrane was subsequently washed with TBST buffer (50 mM Tris, 150 mM NaCl, 0.05% Tween-20, pH 7.5) for 4 times, 5 min each time. DNA was visualized using the Immobilon Western Chemiluniescent HRP Substrate Solution (Millipore, MA, USA) and photographed by Tanon 5200 Multi-Imaging System (Tanon, Shanghai, China).

### RNA extraction and sequencing

Wild-type PAO1 (WT) and a WT::P*rrnB*::*psdR* strain carrying the miniTn7-P*rrnB*-*psdR* were grown in LB-MOPS broth at 37°C overnight and then were diluted to OD_600_ ≈ 0.02 in casamino acids medium. Cells were harvested at an OD_600_ of 2.0. Total RNA was isolated using Eastep Super Total RNA Extraction Kit (Promega, Beijing, China). Total extracted quantity was measured using NanoDrop 1000 spectrophotometer (Thermo, MA, US). Extracted RNA was treated with DNase (RQ1 RNase-free DNase I, Promega, Beijing, China) to remove genomic DNA contamination. The obtained total RNA (2 independent RNA samples per strain) was sent for stranded paired-end mRNA-seq sequencing in Novagen (Tianjin, China) using the Illumina Novaseq Platform.

### Transcriptome analysis

RNA-seq short reads were filtered with a customized Perl script to remove Illumina adapters and low quality of reads (Phred Score, Q < 30). The quality of filtered reads was assessed by FastQC. Trimmed reads were then mapped to the *P*. *aeruginosa* PAO1 reference genome (NC_002516.2) using Hisat2. The SAM files were further converted to BAM files by Samtools. Reads aligned to the reference genome were counted by HTSeq. The generated count files were further processed with the R package DESeq2. Genes were regarded as “differentially expressed” if a fold change ≧ 2.0 and a *P* value ≦ 0.05. The pathway enrichment for differentially expressed genes were conducted using DAVID Bioinformatics Resources (https://david.ncifcrf.gov/) and visualized by GraphPad Prism.

### Transcriptome profile similarity analysis

Published transcriptome datasets of different regulator mutants were downloaded from NCBI SRA database (https://www.ncbi.nlm.nih.gov/sra). The accession numbers of these SRA files are listed in S2 Table. The SRA data files were extracted to FASTQ files using the SRA-tools (version 2.10.4). Customized Perl scripts were then used to filter the short reads with poor quality. Genes exhibiting significant expression changes were identified in the transcriptomes of different regulator mutants using HTSeq (version 0.11.1) and DESeq2. A fold change threshold of ≧2.0 and a *P* value threshold ≦ 0.05 were applied. Subsequently, the common gene set differentially expressed in each regulator mutant was extracted. Next, the expression fold changes of up- or down-regulated common gene set in each regulator mutant transcriptome were used to conduct a one-sided Kolmogorov-Smirnov (KS) test against those of entire gene set exhibiting differential expression in that particular regulator mutant transcriptome. The *P* values generated from KS test were log_10_ transformed and sorted accordingly. Empirical cumulative distribution functions (ECDF) plotting for gene expression changes in each regulator mutant transcriptome was conducted with R software.

### Statistical analysis

Statistical analyses were performed using Excel, GraphPad Prism5 and R software.

### Software

The following software were used in this study.

BWA software, version 0.7.15-r1140 (http://bio-bwa.sourceforge.net) [54];

Cutadapt software, version 1.16 (https://github.com/mafdrcelm/cutadapt/) [55];

Samtools software, version 1.5 (http://samtools.sourceforge.net)[56];

FastQC, version fastqc_v0.11.5 (https://www.bioinformatics.babraham.ac.uk/projects/fastqc/);

Hisat2, version 2.1.0 (https://daehwankimlab.github.io/hisat2/) [57];

HTSeq, version 0.11.1 (https://htseq.readthedocs.io/en/release_0.11.1/count.html) [58];

DESeq2 [59];

Perl software, version v5.22.1 (https://www.perl.org/);

Python software, version v3.8.2 (www.python.org/);

R software, version v3.6.1 (http://www.R-project.org/);

GraphPad Prism software, version 5 (https://www.graphpad.com/);

DAVID Bioinformatics Resources (https://david.ncifcrf.gov/).

## Supporting information

S1 Fig Photograph of strains cultured in casein broth. Photograph of indicated strains grown in casein broth. Strains cultured overnight in LB-MOPS medium were diluted into OD_600_ ≈ 0.02 and subsequently inoculated into casein broth for culturing at 37°C. Photograph was taken after 24 h inoculation. WT, wild-type strain PAO1; Δ*psdR*, PsdR-null mutant; Δ*psdR*Δ*mdpA*Δ*dppA3*, triple gene deletion mutant; Δ*psdR*::P*rrnB*::*psdR*, PsdR-null mutant carrying a single copy of *psdR* driven by a *rrnB* promoter.
(TIF)

S2 Fig The expression of PsdR influences QS activity. P*lasR*-GFP (A), P*rhlA*-GFP (B) and P*pqsA*-GFP (C) reporter plasmids were mobilized into target strains. Strains bearing reporters were grown in casamino acids medium at 37°C. The expression level of GFP in each strain was quantified using a microreader and reported as relative fluorescence units divided by OD_600_. WT, wild-type PAO1; Δ*psdR*, PsdR-null mutant; Δ*psdR*::P*rrnB*::*psdR*, PsdR-null mutant carrying a single copy of *psdR* driven by a *rrnB* promoter. Data of indicated strains are presented as mean ± SD (*n* ≧ 4).
(TIF)

S3 Fig Growth curve of assayed strains. Strains were grown in casamino acids medium. The OD_600_ was measured by microplate reader. The experiment was carried out in eight replicates and the log transformation of mean values is shown.
(TIF)

S4 Fig qRT-PCR analysis of *psdR* expression levels in casamino acids medium and casein broth. (A-B)The wild-type strain PAO1(WT),the PsdR-null mutant (Δ*psdR*) and the PsdR-expressing strain (Δ*psdR*::P*rrnB*::*psdR*) were grown in casamino acids medium or casein broth for 24 h at 37°C. Total RNA of each strain was then extracted for qRT-PCR analysis. Relative expression was normalized using *proC* gene data. Data are means ± SD (3 independent RNA extractions; *n* ≧ 5). *P*-values were obtained from *t*-tests. *P < 0.05, **P < 0.01, ***P < 0.001 (*t*-test).
(TIF)

S5 Fig QS activities were not affected by the deletion of *mdpA* and *dppA3* genes. (A-C) QS activity in PsdR variant derivatives with or without *mdpA* and *dppA3* genes. PsdR variant strains carrying QS reporter plasmids were grown in casein broth for 12 h. Las (A), Rhl (B) and PQS (C) QS activities were estimated by the expression of P*lasR*-GFP, P*rhlA*-GFP and P*pqsA*-GFP reporter plasmids. Fluorescence values were determined using a flow cytometer (FITC channel). (B) Pyocyanin production (OD_695_/OD_600_ values) in shown strains. (C) Cyanide production by these strains. The cyanide-sensitive filter paper was photographed after growth of the bacteria in 24-well plates at 37°C for 18 h. Data are presented as mean ± SD (*n* ≧ 4).
(TIF)

S6 Fig Scheme illustrating the TPSA method. The overall procedure of the TPSA strategy developed in our study. The differentially expressed genes in each transcriptome were first determined. The common differential gene sets was then used to estimate the expression shift of down-regulated or up-regulated genes in each transcriptome by Kolmogorov-Smirnov test (KS test). Transcriptome similarity was assessed according to a ranking of the negative log_10_ transformed *P* values.
(TIF)

S7 Fig PsdR-expressing strain exhibits LasR-null-like phenotypes. (A-C) PsdR-expressing strain produces metabolites to the levels similar to the LasR-null mutant. (A) Pyocyanin production (OD_695_/OD_600_ values) in shown strains. (B) Elastase production (OD_495_/OD_600_ values) in these strains. (C) The relative concentrations of hydrogen cyanide in shown strains. A one-way ANOVA with Bonferroni posttest was used for statistical analysis (n.s., not significant).
(TIF)

S8 Fig PsdR regulates the transcription of PA4677. PsdR regulates the transcription of the PA4677 gene, specifically controlled by LasR. The expression level of PA4677 was estimated with the help of PPA4677-GFP, a PA4677 promoter-GFP fusion construct. Strains carrying PPA4677-GFP were grown in casamino acids medium at 37°C for 12 h. The expression level of GFP in each strain was quantified using a microreader and reported as relative fluorescence units divided by OD_600_. Data of indicated strains are presented as mean ± SD (*n* ≧ 4).
(TIF)

S9 Fig Photograph of strains grown in peptone agar plate. Photograph of indicated strains grown in peptone agar plate. Equal amount of bacteria cultured in LB broth were inoculated onto 2% peptone agar plate and incubated at 37°C for 24 h.
(TIF)

S1 Table Genes differentially expressed in the PsdR-expressing strain. (XLSX)

S2 Table SRA data file used in this study. (XLSX)

S3 Table List of *P*. *aeruginosa* PsdR variant isolates. (XLSX)

S4 Table Combination of DAP-seq and RNA-seq data. (XLSX)

S5 Table Strains used in this study. (XLSX)

S6 Table Oligonucleotides used in this study. (XLSX)

S1 Source Data Source data in this study. (XLSX)
