# Supplementary figures and images for: Uncovering a hidden functional role of the XRE-cupin protein PsdR as a novel quorum-sensing regulator in Pseudomonas aeruginosa
Source: PLoS Pathog. 2024 Mar 14;20(3):e1012078. doi: 10.1371/journal.ppat.1012078 (PMC10965056; doi:10.1371/journal.ppat.1012078)

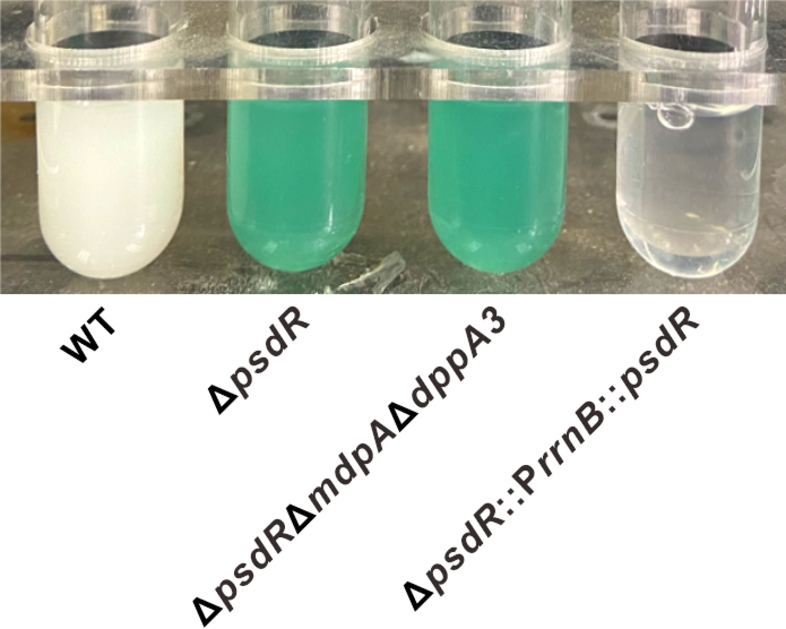

Supplement: S1 Fig — Photograph of indicated strains grown in casein broth. Strains cultured overnight in LB-MOPS medium were diluted into OD600 ≈ 0.02 and subsequently inoculated into casein broth for culturing at 37°C. Photograph was taken after 24 h inoculation. WT, wild-type strain PAO1; ΔpsdR, PsdR-null mutant; ΔpsdRΔmdpAΔdppA3, triple gene deletion mutant; ΔpsdR::PrrnB::psdR, PsdR-null mutant carrying a single copy of psdR driven by a rrnB promoter. (TIF) [file ppat.1012078.s001.tif]

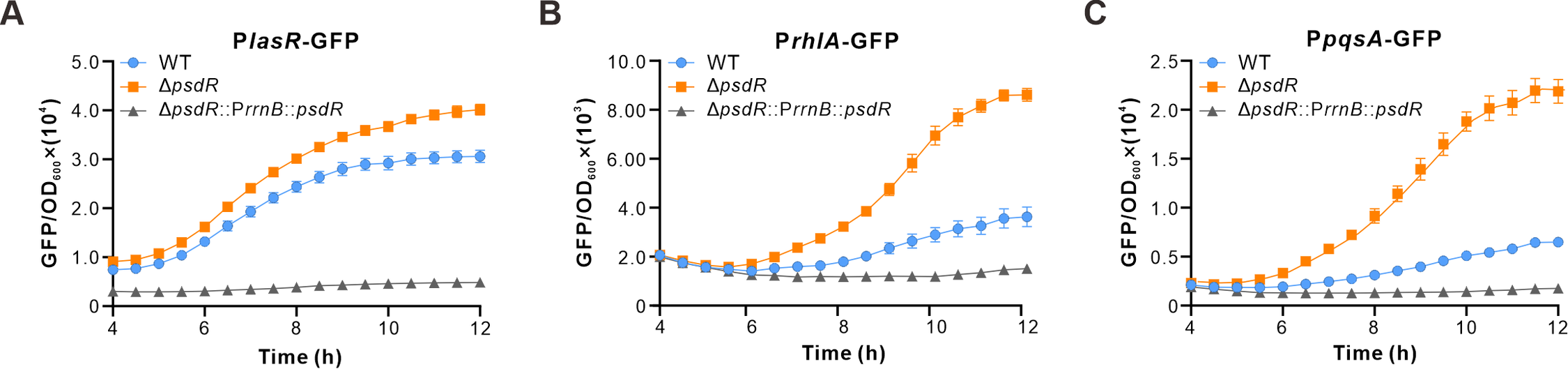

Supplement: S2 Fig — PlasR-GFP (A), PrhlA-GFP (B) and PpqsA-GFP (C) reporter plasmids were mobilized into target strains. Strains bearing reporters were grown in casamino acids medium at 37°C. The expression level of GFP in each strain was quantified using a microreader and reported as relative fluorescence units divided by OD600. WT, wild-type PAO1; ΔpsdR, PsdR-null mutant; ΔpsdR::PrrnB::psdR, PsdR-null mutant carrying a single copy of psdR driven by a rrnB promoter. Data of indicated strains are presented as mean ± SD (n ≧ 4). (TIF) [file ppat.1012078.s002.tif]

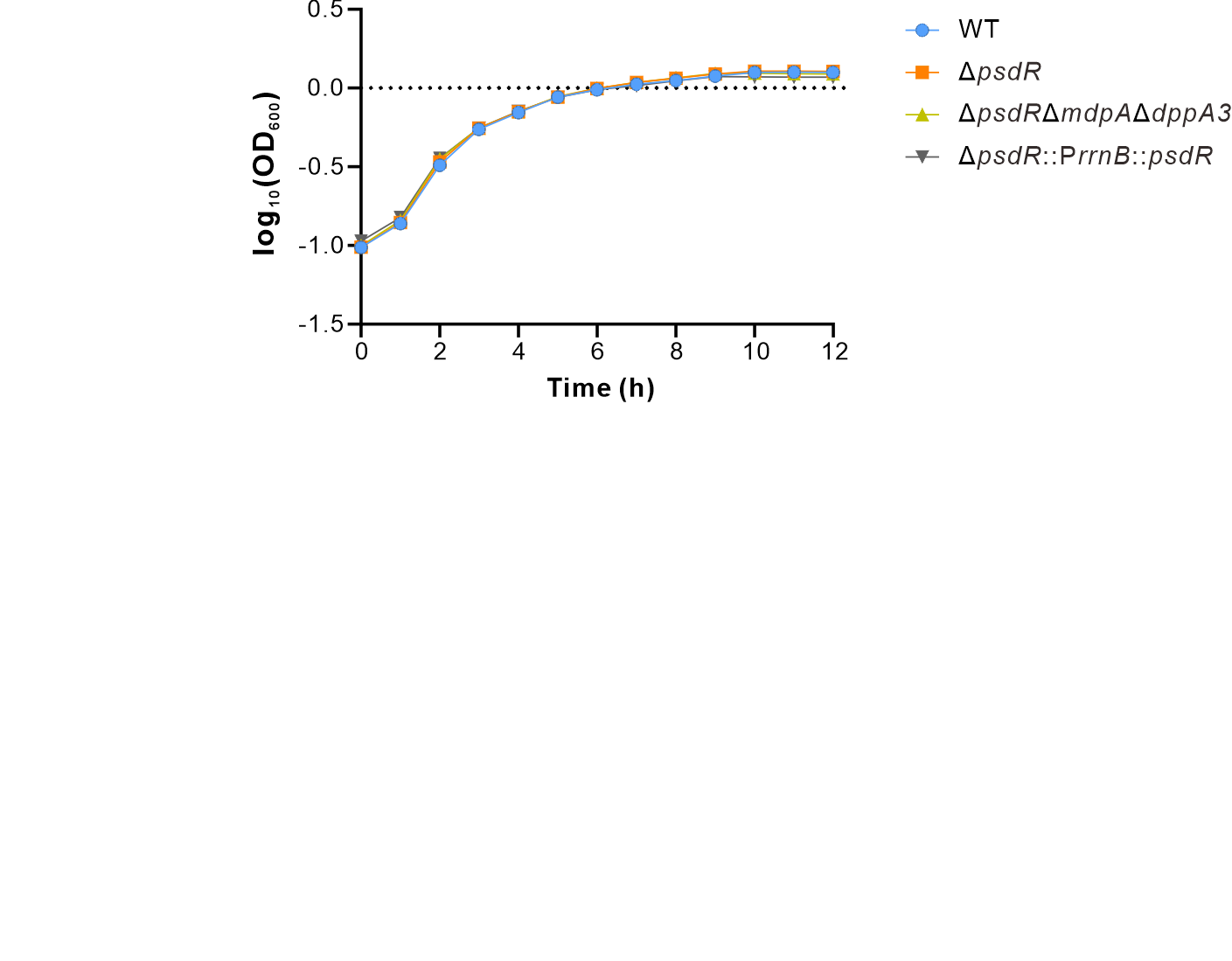

Supplement: S3 Fig — Strains were grown in casamino acids medium. The OD600 was measured by microplate reader. The experiment was carried out in eight replicates and the log transformation of mean values is shown. (TIF) [file ppat.1012078.s003.tif]

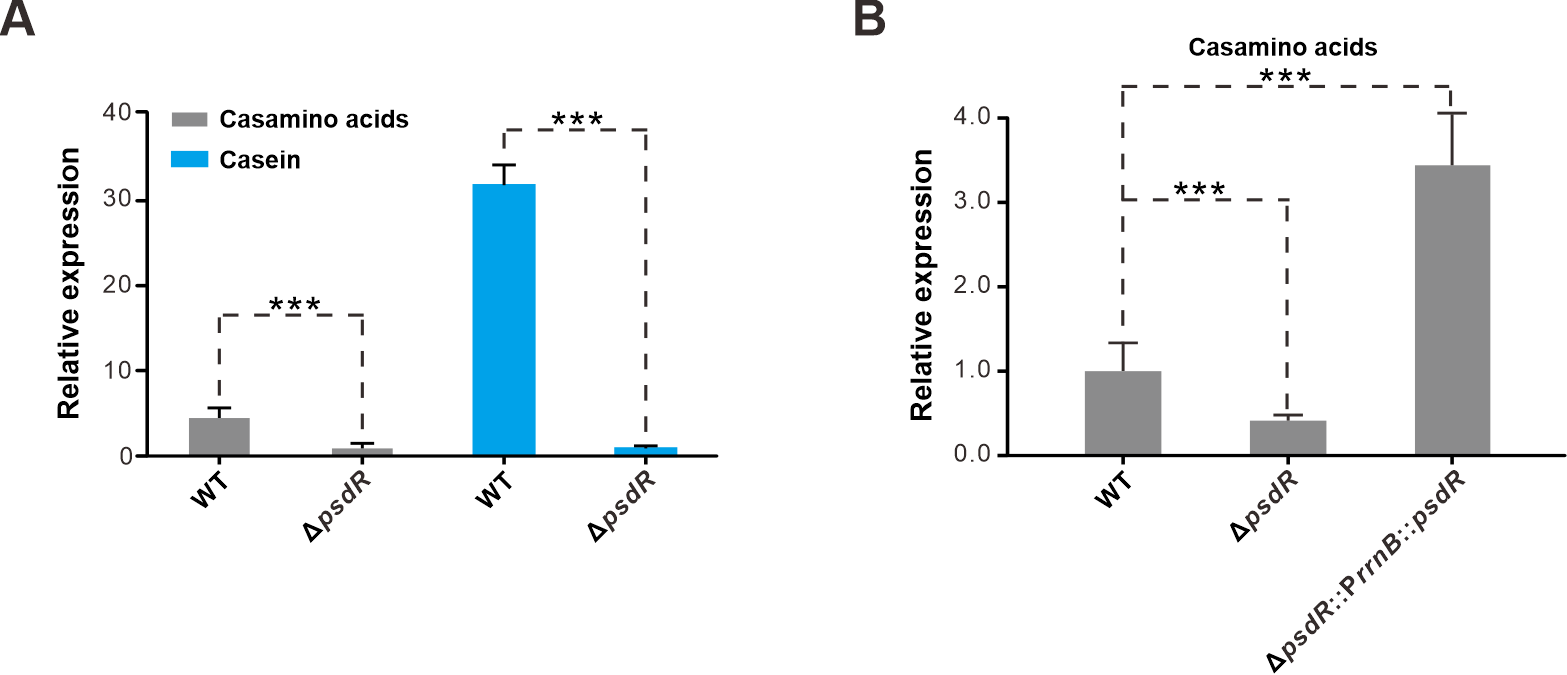

Supplement: S4 Fig — (A-B)The wild-type strain PAO1(WT),the PsdR-null mutant (ΔpsdR) and the PsdR-expressing strain (ΔpsdR::PrrnB::psdR) were grown in casamino acids medium or casein broth for 24 h at 37°C. Total RNA of each strain was then extracted for qRT-PCR analysis. Relative expression was normalized using proC gene data. Data are means ± SD (3 independent RNA extractions; n ≧ 5). P-values were obtained from t-tests. *P < 0.05, **P < 0.01, ***P < 0.001 (t-test). (TIF) [file ppat.1012078.s004.tif]

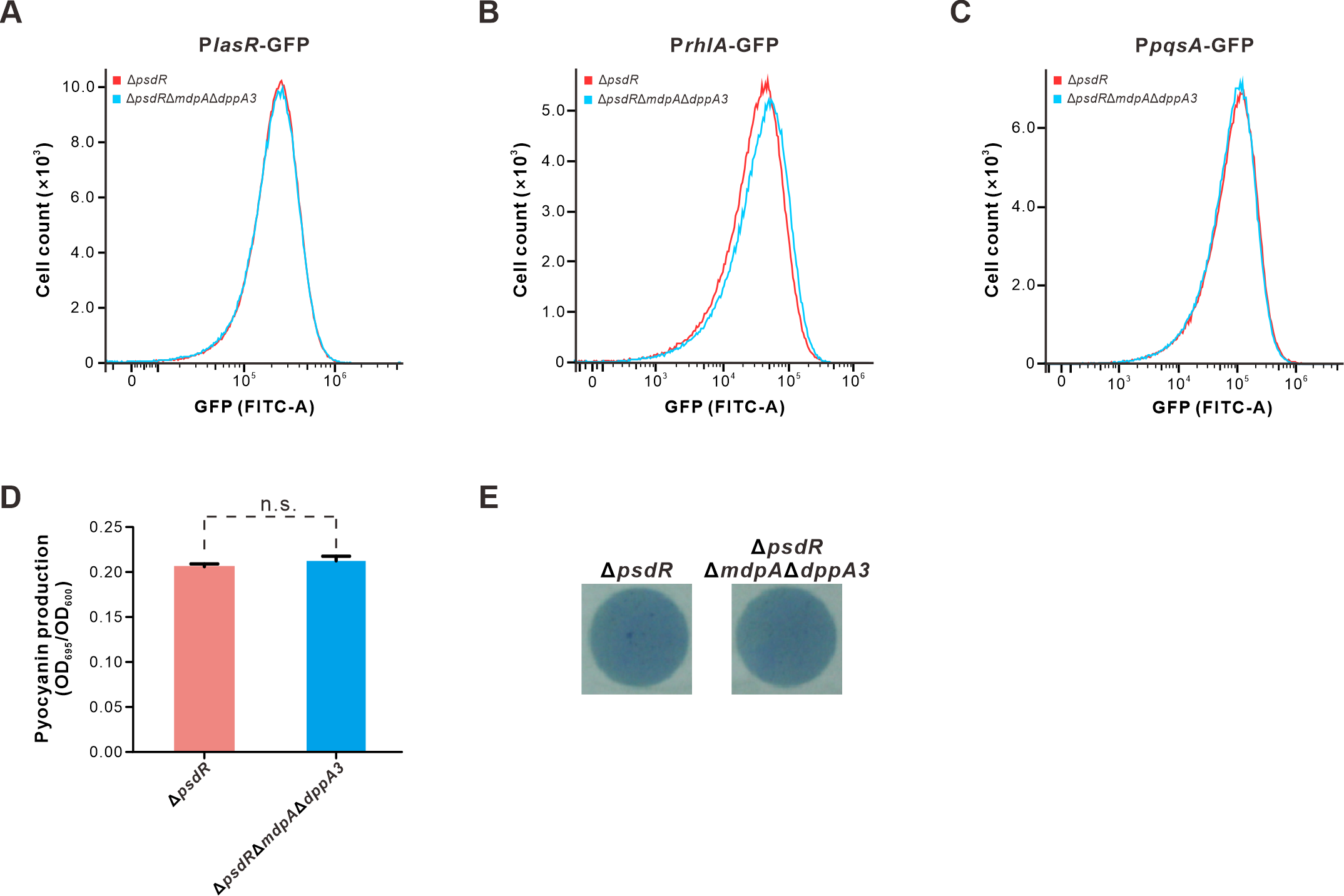

Supplement: S5 Fig — (A-C) QS activity in PsdR variant derivatives with or without mdpA and dppA3 genes. PsdR variant strains carrying QS reporter plasmids were grown in casein broth for 12 h. Las (A), Rhl (B) and PQS (C) QS activities were estimated by the expression of PlasR-GFP, PrhlA-GFP and PpqsA-GFP reporter plasmids. Fluorescence values were determined using a flow cytometer (FITC channel). (B) Pyocyanin production (OD695/OD600 values) in shown strains. (C) Cyanide production by these strains. The cyanide-sensitive filter paper was photographed after growth of the bacteria in 24-well plates at 37°C for 18 h. Data are presented as mean ± SD (n ≧ 4). (TIF) [file ppat.1012078.s005.tif]

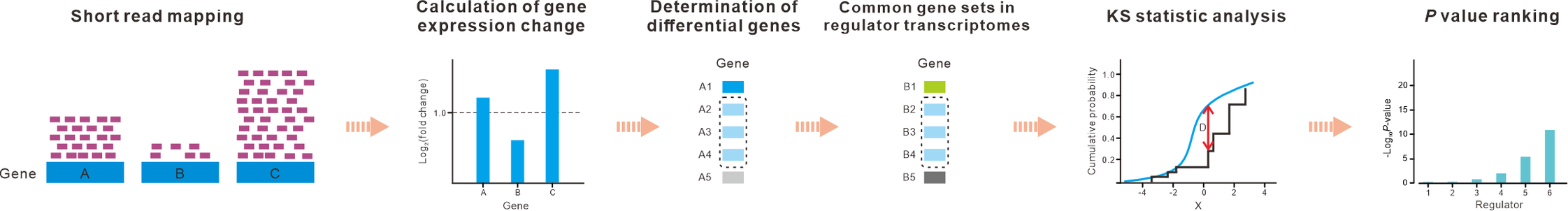

Supplement: S6 Fig — The overall procedure of the TPSA strategy developed in our study. The differentially expressed genes in each transcriptome were first determined. The common differential gene sets was then used to estimate the expression shift of down-regulated or up-regulated genes in each transcriptome by Kolmogorov-Smirnov test (KS test). Transcriptome similarity was assessed according to a ranking of the negative log10 transformed P values. (TIF) [file ppat.1012078.s006.tif]

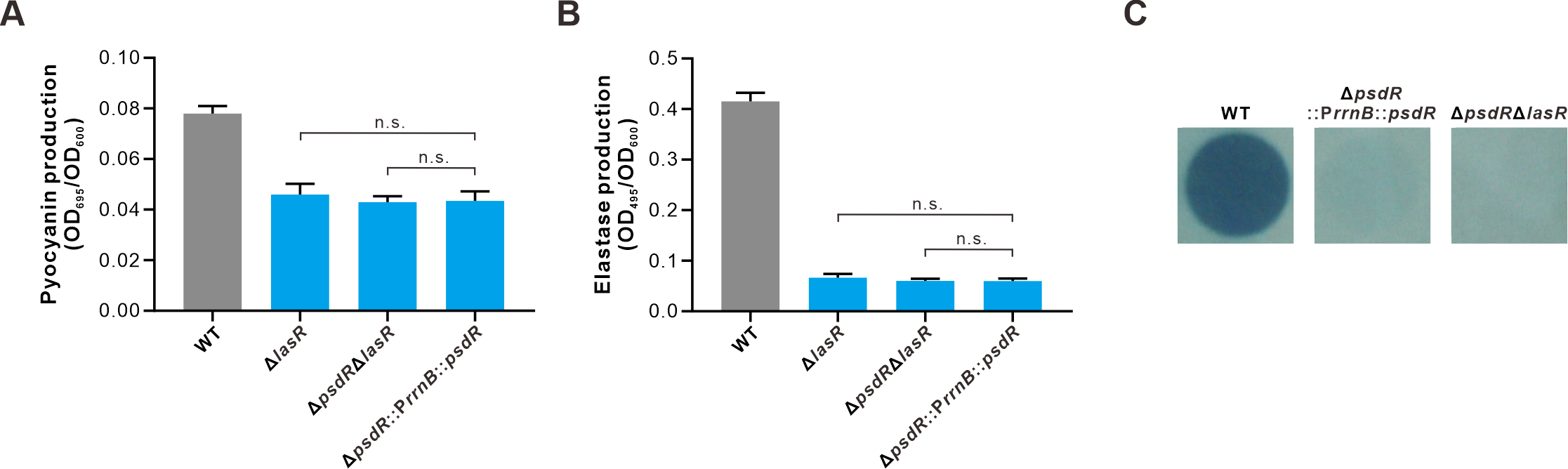

Supplement: S7 Fig — (A-C) PsdR-expressing strain produces metabolites to the levels similar to the LasR-null mutant. (A) Pyocyanin production (OD695/OD600 values) in shown strains. (B) Elastase production (OD495/OD600 values) in these strains. (C) The relative concentrations of hydrogen cyanide in shown strains. A one-way ANOVA with Bonferroni posttest was used for statistical analysis (n.s., not significant). (TIF) [file ppat.1012078.s007.tif]

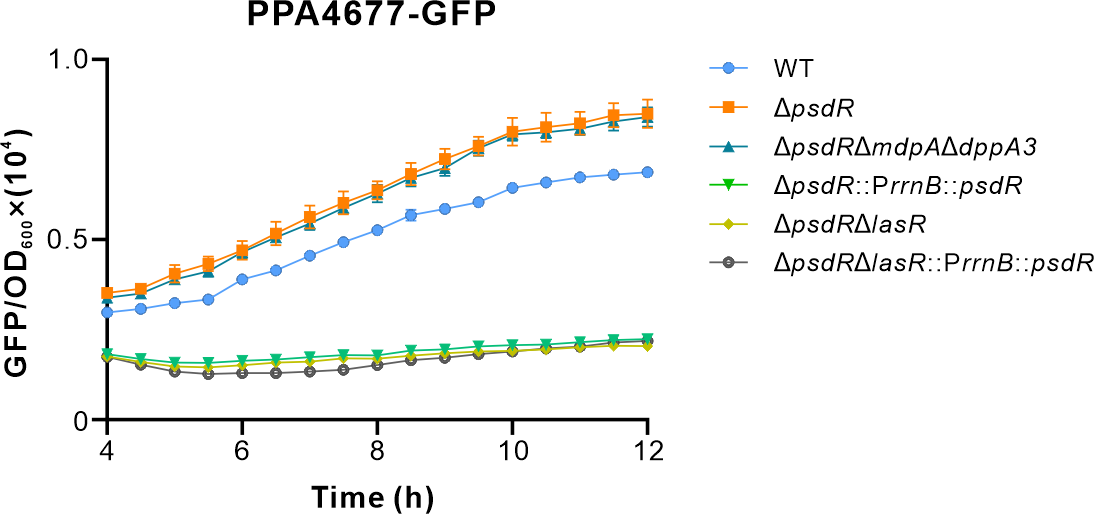

Supplement: S8 Fig — PsdR regulates the transcription of the PA4677 gene, specifically controlled by LasR. The expression level of PA4677 was estimated with the help of PPA4677-GFP, a PA4677 promoter-GFP fusion construct. Strains carrying PPA4677-GFP were grown in casamino acids medium at 37°C for 12 h. The expression level of GFP in each strain was quantified using a microreader and reported as relative fluorescence units divided by OD600. Data of indicated strains are presented as mean ± SD (n ≧ 4). (TIF) [file ppat.1012078.s008.tif]

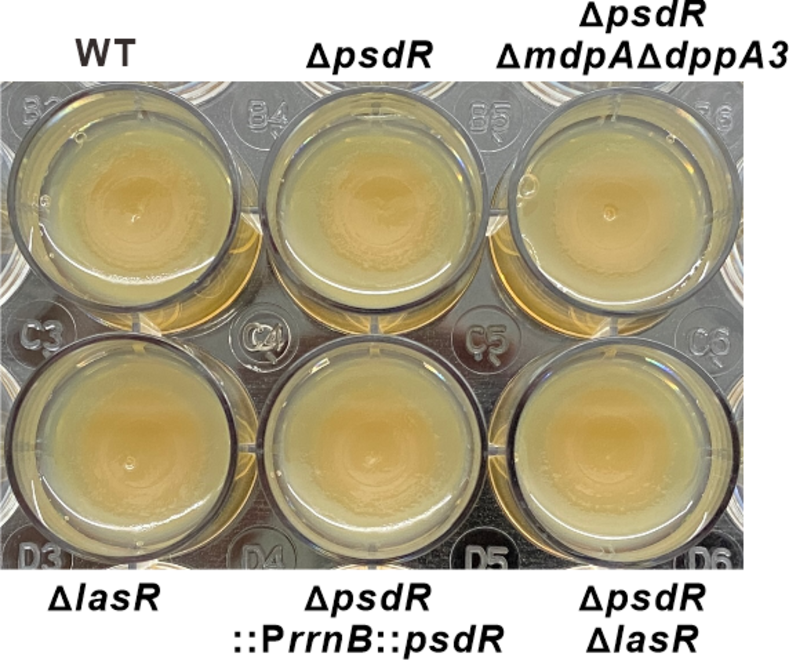

Supplement: S9 Fig — Photograph of indicated strains grown in peptone agar plate. Equal amount of bacteria cultured in LB broth were inoculated onto 2% peptone agar plate and incubated at 37°C for 24 h. (TIF) [file ppat.1012078.s009.tif]
